# Cardiovascular disease outcomes in relation to 25-hydroxyvitamin D and its seasonal variation: Results from the BiomarCaRE consortium

**DOI:** 10.1371/journal.pone.0319607

**Published:** 2025-04-24

**Authors:** Viktor Oskarsson, Veikko Salomaa, Pekka Jousilahti, Luigi Palmieri, Chiara Donfrancesco, Susana Sans, Licia Iacoviello, Simona Costanzo, Marco M. Ferrario, Giancarlo Cesana, Barbara Thorand, Annette Peters, Hugh Tunstall-Pedoe, Mark Woodward, Tanja Zeller, Stefan Blankenberg, Kari Kuulasmaa, Stefan Söderberg

**Affiliations:** 1 Department of Public Health and Clinical Medicine, Umeå University, Umeå, Sweden; 2 Department of Public Health, Finnish Institute for Health and Welfare, Helsinki, Finland; 3 Department of Cardiovascular, Endocrine-Metabolic Diseases and Aging, Istituto Superiore di Sanità-ISS, Rome, Italy; 4 Formerly at the Department of Health, Generalitat of Catalunya, Barcelona, Spain; 5 Research Unit of Epidemiology and Prevention, IRCCS Neuromed, Pozzilli, Italy; 6 Department of Medicine and Surgery, LUM University, Casamassima (Bari), Italy; 7 Department of Medicine and Surgery, University of Insubria, Varese, Italy; 8 Department of Medicine and Surgery, University of Milano Bicocca, Milan, Italy; 9 Institute of Epidemiology, Helmholtz Zentrum München, German Research Center for Environmental Health, Neuherberg, Germany; 10 Institute for Medical Information Processing, Biometry, and Epidemiology (IBE), Ludwig-Maximilians-Universität (LMU), Munich, Germany; 11 German Centre for Cardiovascular Research (DZHK), Partner Site Munich Heart Alliance, Munich, Germany; 12 Institute of Cardiovascular Research, University of Dundee, Dundee, Scotland,; 13 The George Institute for Global Health, Imperial College London, London, United Kingdom; 14 The George Institute for Global Health, University of New South Wales, Sydney, Australia; 15 German Center for Cardiovascular Research (DZHK), partner site Hamburg/Kiel/Luebeck, Hamburg, Germany; 16 University Center of Cardiovascular Science, University Heart and Vascular Center, Hamburg, Germany; 17 Department of Cardiology, University Heart and Vascular Center, Hamburg, Germany; 18 Population Health Research Department, University Heart and Vascular Center, Hamburg, Germany; Public Library of Science, UNITED KINGDOM OF GREAT BRITAIN AND NORTHERN IRELAND

## Abstract

**Background:**

It has been hypothesized but seldom tested that the winter excess in cardiovascular disease (CVD) is related to hypovitaminosis D. The present study examined the association between CVD and (i) seasonality of 25-hydroxyvitamin D (25[OH]D) and (ii) individual 25(OH)D concentrations.

**Methods and findings:**

Harmonized 25(OH)D data were obtained from the Biomarkers for Cardiovascular Risk Assessment in Europe (BiomarCaRE) project, including 79,570 participants examined between 1984 and 2010. One 25(OH)D measurement was available per participant. Primary endpoints were CVD incidence (coronary heart disease or stroke; *n* = 6006) and CVD mortality (*n* = 2985). To study (i), Poisson regression-derived rate ratios were compared according to two-month categories, ordered by baseline 25(OH)D concentrations. To study (ii), Cox regression-derived hazard ratios were compared according to quarters of baseline 25(OH)D concentrations. With respect to (i), despite a median 25(OH)D concentration ratio of 1:1.79, the trough months of 25(OH)D in March and April had a similar CVD incidence as the peak months of 25(OH)D in August and September (rate ratio: 1.07, 95% CI: 0.98–1.17). CVD mortality was slightly higher in the trough months compared to the peak months (rate ratio: 1.27, 95% CI: 1.12–1.44) but not compared to the other months (despite median 25[OH]D concentration ratios up to 1:1.62; *p* ≥ 0.077). The CVD mortality peak in January preceded the 25(OH)D trough, not adhering to the temporality criterion of Bradford Hill. With respect to (ii), compared to the lowest quarter, the highest quarter of 25(OH)D was associated with lower CVD incidence (hazard ratio: 0.82, 95% CI: 0.76–0.89) and CVD mortality (hazard ratio: 0.64, 95% CI: 0.57–0.72).

**Conclusion:**

The present study does not support the hypothesis that seasonal increases in CVD are driven by short-term reductions in 25(OH)D. As in most observational studies, higher 25(OH)D concentrations were inversely associated with CVD.

## Introduction

Spurred by the inverse association of 25-hydroxyvitamin D (25[OH]D) status with a large number of cardiovascular diseases (CVD) in observational data [1–7], the role of 25(OH)D in the primary and secondary prevention of CVD has been heavily debated during the last decades. The initial enthusiasm has, however, been lessened by the inconsistent findings from Mendelian randomization studies on the association between genetically-predicted 25(OH)D concentrations and CVD outcomes [8–15] and by the null findings from randomized clinical trials on the association between vitamin D supplementation and CVD outcomes [16–20].

25(OH)D concentrations are largely affected by sunlight exposure [21], leading to a seasonal difference between late summer and early spring that might exceed 100% [22,23]. In 1981, in response to the long-lasting observation of a winter excess in CVD outcomes [24,25], it was hypothesized by Scragg that the seasonal variability in CVD was driven by hypovitaminosis D [26]. This ecological hypothesis stood untested until 2015, when the following seasonality postulates were tested in the Scottish Heart Health Extended Cohort (SHHEC) [27]: (i) outcome variability is inversely associated with the seasonal fluctuation in 25(OH)D and (ii) outcome variability is accentuated in persons with “low” 25(OH)D concentrations, since they should experience more hypovitaminosis D during the winter. Both of those postulates were rejected in the SHHEC, thereby questioning a causal link between short-term changes in 25(OH)D and seasonality of CVD. First, there was no peak or trough in CVD incidence and the peak and trough in CVD mortality occurred near the solstices, preceding the extremes of 25(OH)D by at least two months. Second, there was no increased CVD susceptibility during the 25(OH)D trough in persons with below-median 25(OH)D concentrations. To the best of our knowledge, no subsequent study has examined the seasonal variation of CVD in relation to that of 25(OH)D.

Using harmonized 25(OH)D data from almost 80,000 participants in the Biomarkers for Cardiovascular Risk Assessment in Europe (BiomarCaRE) project, we conducted a detailed analysis on the association of CVD outcomes with seasonal variation in 25(OH)D and with individual 25(OH)D concentrations among different latitude populations.

## Materials and methods

Writing and reporting was conducted in accordance with the Strengthening the Reporting of Observational Studies in Epidemiology (STROBE) statement (S1 Checklist) [28].

### Study population

The BiomarCaRE project, which is an extension of the Monitoring of Trends and Determinants in Cardiovascular Disease (MONICA) Risk, Genetics, Archiving, and Monograph (MORGAM) project [29,30], was established in the early 2010s with the purpose to harmonize between-cohort data on CVD biomarkers [31]. Eight BiomarCaRE cohorts have been subjected to 25(OH)D measurements and were included in the present study (i.e., MONICA Northern Sweden, FINRISK 1997, SHHEC, MONICA/Cooperative Health Research in the Region of Augsburg [KORA], MONICA Brianza, Malattie Aterosclerotiche Istituto Superiore di Sanità [MATISS], Moli-sani, and MORGAM/MONICA-Catalonia; see S1 Table for detailed cohort descriptions).

#### Ethical considerations.

Written informed consent was obtained from all study individuals. The MONICA Northern Sweden study was approved by the Regional Ethical Committee at Umeå University, Sweden. The FINRISK 1997 study was approved by the Ethical Committee of the National Public Health Institute, Finland. The SHHEC was approved by the then Privacy Advisory Committee and Chief Scientist Committee of the Scottish Home and Health Department and, subsequently, by approximately 30 individual local research ethics committees in Scotland. The MONICA/KORA study was approved by the local authorities in southern Germany and conducted in accordance with the data protection regulations. The KORA study was further approved by the Ethics Committee of the Bavarian Chamber of Physicians, Germany. The MONICA Brianza study was approved by the Comitato Etico Azienda Ospedaliera San Gerardo-Monza, Italy. The Moli-sani study was approved by the Rome Catholic University Ethical Committee, Italy. The MATISS study was approved by the Ethical Committee of the Istituto Superiore di Sanità-ISS, Italy. The MORGAM/MONICA-Catalonia study was approved by the Board of the former Institute of Health Studies, Department of Health and Social Security, Generalitat of Catalunya, Spain.

Additional information regarding the ethical, cultural, and scientific considerations specific to inclusivity in global research is included in the Supporting information (S2 Checklist).

### Exposure and covariate assessment

**Fig 1:**
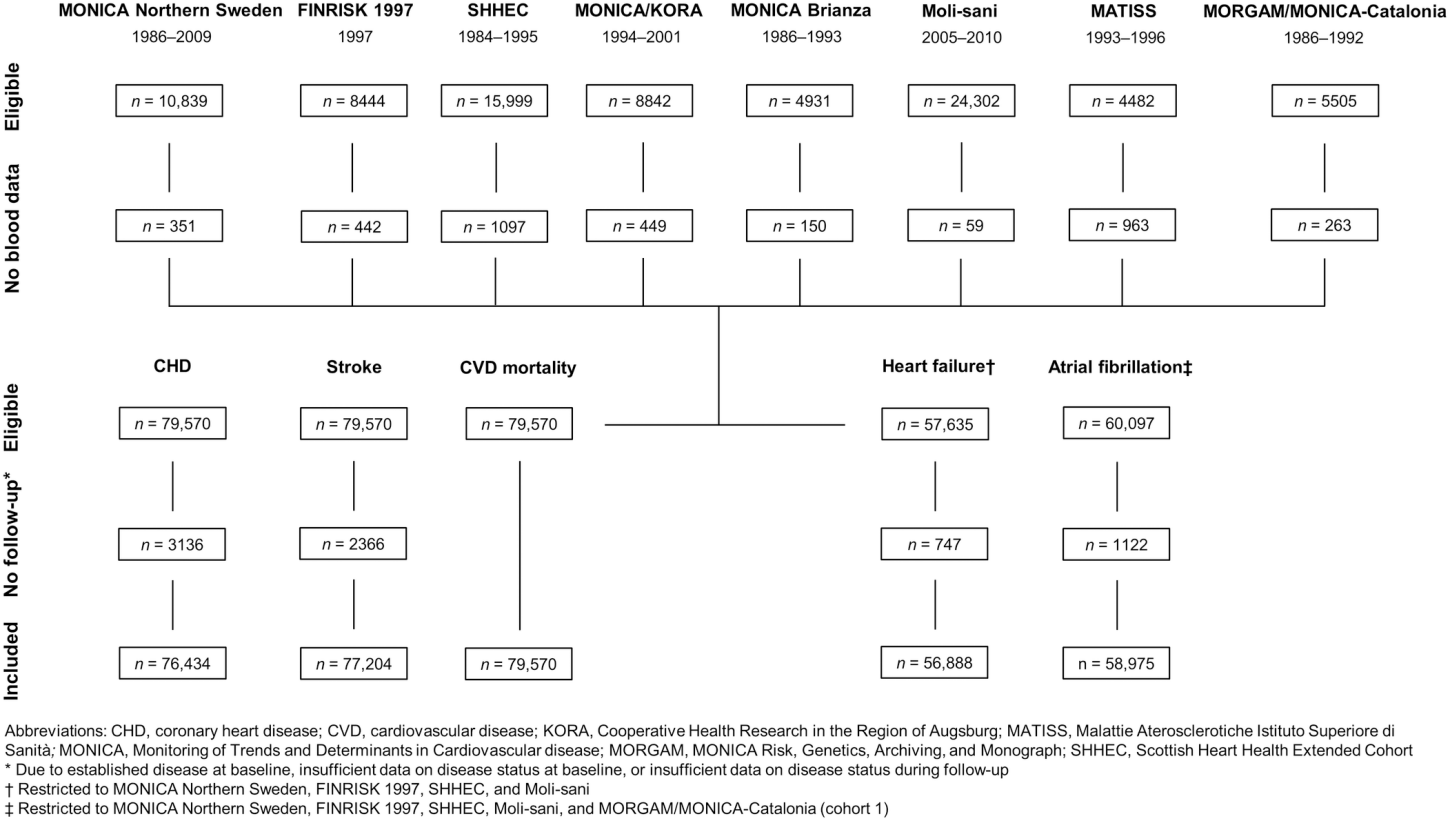
Participant flow chart. The calendar years specified below each cohort name indicate the time period for the baseline examinations.

Details of the harmonized 25(OH)D analyses within the BiomarCaRE project have been published elsewhere [22] and are outlined in S1 Text. In brief, we used a one-step immunoassay on the ARCHITECT i2000 system (Abbott Diagnostics, Abbott Park, IL, USA) [32]. A single 25(OH)D measurement was available for each participant and collected between 1984 and 2010 (Fig 1).

The following harmonized covariates were available at baseline: age, sex, education, body mass index (BMI), smoking status, systolic and diastolic blood pressure, antihypertensive medication, total cholesterol concentration, creatinine-estimated glomerular filtration rate (crea-eGFR; based on the Chronic Kidney Disease Epidemiology Collaboration equation shown in S1 Text), N-terminal pro-B-type natriuretic peptide concentration, high-sensitive troponin I concentration, and history of diabetes, coronary heart disease, stroke, heart failure, atrial fibrillation, and diabetes. (Nota bene: Data on education were not collected in MATISS, data on prevalent heart failure were not collected in MONICA Brianza and MATISS, and data on prevalent atrial fibrillation were not collected in MONICA/KORA, MONICA Brianza, and MATISS.)

### Endpoint assessment

CVD endpoints (i.e., coronary heart disease, stroke, heart failure, atrial fibrillation, and CVD mortality) were measured in each cohort by standardized follow-up procedures (visit, report, register, etc.; S1 Table). Definitions of the endpoints, including their availability by cohort at baseline and during follow-up, are shown in S2 Table. Coronary heart disease incidence, stroke incidence, and CVD mortality were assessed in all cohorts, while heart failure incidence and atrial fibrillation incidence were assessed in a subset of cohorts (Fig 1). Due to their cohort-wide availability, the primary endpoints were (i) a composite of coronary heart disease incidence and stroke incidence (hereinafter referred to as CVD incidence) and (ii) CVD mortality. Incident cases of coronary heart disease, stroke, heart failure, and atrial fibrillation were used as secondary endpoints. Prevalent cases of each endpoint at baseline were excluded from the incidence analyses but not from the mortality analyses.

### Statistical analysis

Statistical significance was set at a two-sided *P*-value less than 0.05 and all analyses were performed in Stata version 14 (StataCorp LP, College Station, TX, USA). The statistical code is available from the corresponding author upon request.

#### Cross-sectional analyses.

A total of 79,570 participants were eligible for cross-sectional analyses (Fig 1). To study the cohort-specific associations between 25(OH)D status and covariates, we used quantile regression models adjusted for sex, age (continuous using four-knot restricted cubic splines at the fifth, 35th, 65th, and 95th percentile, years), and calendar season of sampling (winter, spring, summer, and fall).

#### Study populations and follow-up data in prospective analyses.

The number of eligible participants in time-to-event analyses varied by endpoint and ranged from 56,888 participants in the heart failure analysis to 79,570 participants in the CVD mortality analysis (Fig 1). Time-at-risk for an incident endpoint was censored at the date of that endpoint, the date of death, or the end of the follow-up period (with separate models for each endpoint). For composite endpoints, time-at-risk was censored at the date of the first endpoint (if multiple endpoints had occurred), the date of death, or the end of the shortest follow-up period (if endpoints had different follow-up periods, as they had in Moli-sani and MONICA/KORA). Time-at-risk for CVD mortality was censored similarly, ignoring non-fatal endpoints.

#### Seasonality of CVD outcomes and 25(OH)D status.

Poisson regression models were used to calculate the incidence and mortality rates of CVD by calendar month. A detailed description of the case and person-year calculation is given in S1 Text, but, in brief, we split the follow-up time for each participant on the last day of each calendar month throughout the entire follow-up period (creating up to 12 million observations, depending on the endpoint). To examine whether the seasonal variation in CVD corresponded with that of 25(OH)D, we then calculated sex-, attained age-, and cohort-adjusted rate ratios according to two-month calendar periods. The calendar periods were, in turn, ranked from highest to lowest according to baseline 25(OH)D concentrations using the sex- and age-adjusted median values: August, September, July, October, November, June, December, January, May, February, March, and April. As such, the 25(OH)D ranking of calendar months was based on cross-sectional rather than prospective data (see S1 Fig for unadjusted 25[OH]D data). In sensitivity analyses, the calendar periods were re-arranged according to three-, four-, and six-month groups. Subgroup analyses were performed on persons with “low” and “high” baseline 25(OH)D concentrations, using the sex-, cohort-, and calendar month-specific median value as cut-off value, with the purpose to test for a heterogeneous seasonal variation of CVD outcomes by individual 25(OH)D concentrations. Subgroup analyses were also performed by attained age (≤70/>70 years), sex (men/women), and cohort (eight levels) (S1 Text).

#### Association between 25(OH)D exposure and CVD outcomes.

Cox regression models, with attained age as the time scale, were used to calculate hazard ratios (HRs) of CVD endpoints by quarters of individual 25(OH)D concentrations and, in a sensitivity analysis, by tenths of individual 25(OH)D concentrations. To account for within- and between-cohort differences in the calendar month of blood sampling, as well as the very large variation in 25(OH)D by sunlight exposure, the categories of 25(OH)D were sex-, cohort-, and calendar month-specific. We chose this methodology since it is reasonable to assume that individuals who are (i) ranked high according to percentiles during the winter also are ranked high during the summer and (ii) of the same sex and from the same geographical area are likely to have a similar sunlight exposure. For the same reasons, 25(OH)D status was not analyzed as a continuous variable. The standard assumption of proportional hazards along the time scale was evaluated by modeling the possible interaction between the time scale and the categorical exposure variable (S1 Text) [33].

The basic model was adjusted for sex (men/women), cohort (eight levels), and calendar week of blood sampling (continuous). The multivariable model was further adjusted for BMI (continuous, kg/m^2^), smoking status (no/yes), systolic blood pressure (continuous, mmHg), antihypertensive medication (no/yes), total cholesterol concentration (continuous, mmol/L), crea-eGFR (continuous, ml/min/1.73 m^2^), history of diabetes (no/yes), and history of CVD (no/yes; only included in the mortality analysis). Continuous covariates were modeled using four-knot restricted cubic splines at the fifth, 35th, 65th, and 95th percentile. Sex, smoking status, antihypertensive medication, and history of diabetes and CVD were accounted for by stratification of the baseline hazard function, because they did not always meet the proportional hazards assumption.

Missing data on 25(OH)D and covariates in the primary endpoint analyses were handled using multiple imputation by chained equations. Endpoint data were not imputed. The percentages of missing data by endpoint and analytical model are shown in S3 Table, and a detailed description of the multiple imputation method is reported in S1 Text. In a sensitivity analysis, we repeated the analysis using complete case data. In a first extension of the complete case model, in which participants from MATISS and subcohort 4 of MONICA/KORA were excluded due to lack of education data (*n* = 6924 and 7559 participants for CVD incidence and mortality, respectively), we further adjusted the multivariable model for education (three levels). In further extensions of the complete case model, which aimed to account for exposure misclassification over time and for long baseline examination periods in some cohorts, we restricted the follow-up time to a maximum of five years and re-categorized the exposure variable according to calendar year of examination (S1 Text). To avoid repeated imputations—and since results based on imputed and complete case data were almost identical for CVD incidence and mortality—the Cox regression models of secondary endpoints were only based on complete case data.

To examine reverse causality bias, we constructed three separate models with different baseline exclusions in another sensitivity analysis (S1 Text). Separate models by sex (men/women) and cohort (eight levels) were also performed as subgroup analyses (S1 Text).

## Results

### Cross-sectional analyses

A total of 79,570 participants from eight cohort studies were included for cross-sectional analyses (48.9% men; median age 50.7 years; examination period 1984–2010) (Fig 1). Baseline characteristics by cohort are presented in S4 Table. Most cohorts had examined participants throughout the calendar year, except in the Swedish and Finnish cohort, which had examined participants almost exclusively from winter to spring. The median 25(OH)D concentration ranged from 32.4 nmol/L in Finland (due to the winter-to-spring examination period) to 45.2 nmol/L in Sweden (despite the winter-to-spring examination period).

The cohort-specific associations between 25(OH)D status and other variables are shown in Table 1. Older participants had markedly lower 25(OH)D concentrations than younger participants in Germany, Italy, and Spain; in contrast, the opposite was found in Sweden and Finland (see S2 Fig for the cohort-specific shape of the 25[OH]D-by-age association). Except in the Swedish cohort, male participants had higher 25(OH)D concentrations than female participants. Examination during late summer and early autumn led to strikingly higher 25(OH)D concentrations, with a peak-to-trough swing of 79–185% in cohorts with a four-season examination period. In general, 25(OH)D status had an inverse association with smoking and obesity and a positive association with education. Prevalent CVD, hypertension, diabetes, and hypercholesterolemia were associated with lower 25(OH)D concentrations, while prevalent chronic kidney disease was associated with higher 25(OH)D concentrations (see S3 Fig for a detailed analysis of the 25[OH]D-by-kidney function association).

**Table 1:** Sex-, age-, and season-adjusted median difference^a^ in 25(OH)D status (nmol/L) by baseline characteristics in the study population with complete data on 25(OH)D (*n* = 75,808) and according to cohort^b^^.^

	Cohort							
Characteristics	MONICA Northern Sweden	FINRISK 1997	SHHEC	MONICA/ KORA	MONICA Brianza	Moli-sani	MATISS	MORGAM/MONICA-Catalonia
Age (90th vs. tenth percentile)^c^	**3.22**	**12.10**	0.19	**-7.20**	**-11.24**	**-8.81**	**-18.73**	**-4.89**
Male vs. female sex	**-1.34**	**2.88**	**4.47**	**4.82**	**6.61**	**8.14**	**13.13**	**3.87**
Calendar month (peak vs. trough)^d^	**8.72**	**2.34**	**27.18**	**34.67**	**24.37**	**30.68**	**43.76**	**29.09**
Highest vs. lowest third of education^e^	0.26	**1.82**	**2.19**	**2.42**	-0.32	0.50	—	**2.95**
BMI ≥ 30 vs. <25 kg/m^2^	**-9.78**	**-3.77**	**-2.81**	**-5.68**	**-3.40**	**-5.27**	-1.89	**-2.48**
Daily vs. non-daily smoking	**-1.18**	**-2.50**	**-6.05**	**-5.16**	**-3.75**	**-4.77**	**-4.42**	**-3.29**
Comorbidities (yes vs. no)								
Cardiovascular disease^f^	-1.73	-1.05	**-2.31**	**-3.50**	**-5.31**	**-3.73**	-0.50	0.12
Hypertension^g^	**-1.58**	**-0.90**	**-1.60**	**-2.69**	-**1.52**	**-3.49**	-1.49	**-1.87**
Diabetes	**-1.99**	-1.94	**-3.53**	**-2.71**	1.07	**-3.04**	**-3.80**	**-1.81**
Hypercholesterolemia^h^	0.89	**-4.43**	**-3.47**	**-3.80**	**-5.17**	**-2.84**	-0.62	-1.19
CKD stage two to five^i^	**12.63**	**2.39**	**2.39**	**4.01**	**7.16**	**1.70**	**5.14**	**7.44**

25(OH)D, 25-hydroxyvitamin D; BMI, body mass index; CKD, chronic kidney disease; KORA, Cooperative Health Research in the Region of Augsburg; MATISS, Malattie Aterosclerotiche Istituto Superiore di Sanità*;* MONICA, Monitoring of Trends and Determinants in Cardiovascular disease; MORGAM, MONICA Risk, Genetics, Archiving, and Monograph; SHHEC, Scottish Heart Health Extended Cohort

^a^Estimated from quantile regression models and adjusted for sex, age (continuous using four-knot restricted cubic splines, years), and season of sampling (winter, spring, summer, and fall). Bold text denotes statistically significant findings (p < 0.05). The number of participants in each cohort is shown in S4 Table. All calculations were based on complete data (see S3 Table for cohort-specific percentages of missing data)

^b^The number of participants with complete data on 25(OH)D status were 10,405 in MONICA Northern Sweden, 7787 in FINRISK 1997, 13,224 in SHHEC, 8023 in MONICA/KORA, 4488 in MONICA Brianza, 23,433 in Moli-sani, 3443 in MATISS, and 5005 in MORGAM/MONICA-Catalonia

^c^Except in MONICA Brianza, SHHEC, and MORGAM/MONICA-Catalonia (p for non-linearity = 0.17, 0.06, and 0.11, respectively), there was evidence of non-linear associations between 25(OH)D status and age in all cohorts (p for non-linearity < 0.05; obtained via the Wald test, testing the second and third spline transformation jointly equal to zero)

^d^Comparing the peak month of 25(OH)D status with the trough month of 25(OH)D status. The Swedish and Finnish participants were almost exclusively examined between January and April, leading to a small seasonal swing

^e^Categories of education were derived from population-, sex-, and birth cohort-specific thirds of the distribution of years of education. No data on education were available in MATISS and in subcohort 4 of MONICA/KORA (*n* = 4005)

^f^Coronary heart disease or stroke

^g^Systolic blood pressure > 140 mmHg, diastolic blood pressure > 90 mmHg, or use of antihypertensive medication

^h^Total cholesterol concentration > 7.0 mmol/L

^i^Creatinine-estimated glomerular filtration rate < 90 ml/min/1.73 m^2^

### Prospective analyses

#### Study populations and follow-up data.

As can be seen in Fig 1, the number of included cohorts in time-to-event analyses varied by CVD endpoint. The total number of participants, person-years, and cases for each endpoint are shown in Table 2. The incidence rate per 10,000 person-years ranged from 54.7 cases for coronary heart disease to 24.6 cases for stroke; and the mortality rate was 30.0 cases per 10,000 person-years. (Nota bene: The cohort-specific incidence and mortality rates are given in S5 Table, conditional on age, sex, and decade; and the co-occurrence of endpoints is presented S6 Table.)

**Table 2:** Follow-up data in time-to-event analyses.

	Follow-up data			
Endpoint	No. of participants	No. of person-years^a^	No. of cases^a^	Crude rate^b^
Incidence				
Coronary heart disease^c^	76,434	845,157	4619	54.7 (53.1 to 56.3)
Stroke^c^	77,204	952,626	2345	24.6 (23.6 to 25.6)
Heart failure	56,888	719,060	3226	44.9 (43.3 to 46.4)
Atrial fibrillation	58,975	739,365	2848	38.5 (37.1 to 40.0)
Mortality				
Cardiovascular disease	79,570	994,145	2985	30.0 (29.0 to 31.1)

^a^The number of cohort-specific person-years and cases ranged from:

Coronary heart disease: 269,785–33,447 person-years (Scottish Heart Health Extended Cohort [SHHEC] and Malattie Aterosclerotiche Istituto Superiore di Sanità [MATISS], respectively) and 2117–79 cases (SHHEC and MATISS, respectively)

Stroke: 281,674–33,699 person-years (SHHEC and MATISS, respectively) and 845 to seven cases (SHHEC and Monitoring of Trends and Determinants in Cardiovascular disease [MONICA] Risk, Genetics, Archiving, and Monograph [MORGAM]/MONICA-Catalonia, respectively)

Heart failure: 286,810–100,514 person-years (SHHEC and FINRISK 1997, respectively) and 1201–458 cases (Moli-sani and MONICA Northern Sweden, respectively)

Atrial fibrillation: 285,511–23,186 person-years (SHHEC and MORGAM/MONICA-Catalonia, respectively) and 1045–12 cases (Moli-sani and MORGAM/MONICA-Catalonia, respectively)

Cardiovascular disease mortality: 290,813–34,208 person-years (SHHEC and MATISS, respectively) and 1547–42 cases (SHHEC and MORGAM/MONICA-Catalonia, respectively)

^b^Per 10,000 person-years (95% CI)

^c^When using coronary heart disease and stroke as a composite endpoint, the number of participants was 74,867, the number of person-years was 821,774, the number of cases was 6006, and the crude rate was 73.1 (95% CI: 71.3–75.0)

#### Seasonality of CVD outcomes and 25(OH)D status.

**Fig 2:**
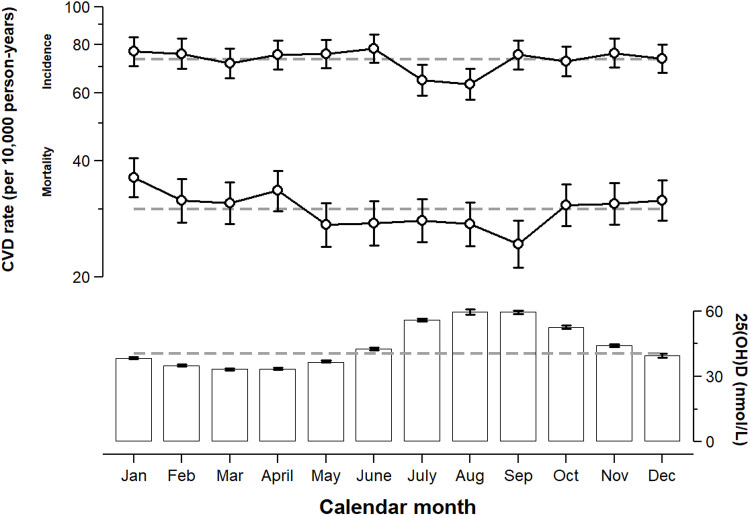
Seasonal variation of (i) cardiovascular disease (CVD) incidence and mortality during follow-up and (ii) 25-hydroxyvitamin D (25[OH]D) at baseline. Hollow circles and capped spikes represent point estimates and 95% CI for Poisson regression-derived rates (left y-axis). Bars and capped spikes represent point estimates and 95% CI for quantile regression-derived median concentrations (right y-axis). The dashed lines represent point estimates for the entire calendar year.

There was a large difference in the median 25(OH)D concentration by calendar month, with the peak observed in August and the trough observed in March (+48 and -18%, respectively, compared to the full year estimate) (Fig 2). For CVD incidence, the observed rates were rather stable over the calendar year except for a trough in July and August (trough and peak difference of -14 and +7% compared to the full year estimate). A more variable pattern was observed for CVD mortality, with a trough that started in May and reached a minimum in September and with a peak in January (trough and peak difference of -19 and +21% compared to the full year estimate).

Sex-, attained age-, and cohort-adjusted rate ratios of CVD incidence and mortality by two-month calendar periods, ordered by 25(OH)D status, are shown in Table 3 (and by three- to six-month calendar periods in S7 Table). The trough months of 25(OH)D in March and April had a similar incidence but a higher mortality of CVD than the peak months of 25(OH)D in August and September (*p* for comparison = 0.12 and < 0.001, respectively). There were no differences in CVD outcomes between the trough months and the non-peak months of 25(OH)D (despite median concentration differences up to 62%; *p* for comparison ≥ 0.077). (Nota bene: Incidence rate ratios for coronary heart disease and stroke according to the same categories are presented in S8 Table, for which there was a difference between the trough and peak months of 25[OH]D for stroke but not for coronary heart disease.)

**Table 3:** Rate ratios of CVD incidence and mortality by two-month calendar periods (ordered by 25[OH]D status).

	Two-month calendar period (ordered by increasing differences in 25[OH]D concentration)^a^					
	August, September	July, October	June, November	January, December	February, May	March, April
CVD incidence^b^						
No. of cases	957	966	1051	1041	995	996
No. of person-years	138,484	140,938	136,507	138,615	131,493	135,943
Rate ratio (95% CI)^c^	1.00 (reference)	0.99 (0.91 to 1.09)	1.12 (1.02 to 1.22)	1.09 (1.00 to 1.19)	1.11 (1.01 to 1.21)	1.07 (0.98 to 1.17)
CVD mortality						
No. of cases	434	501	484	568	467	531
No. of person-years	167,434	170,389	165,020	167,815	159,194	164,510
Rate ratio (95% CI)^c^	1.00 (reference)	1.14 (1.00 to 1.29)	1.13 (1.00 to 1.29)	1.31 (1.16 to 1.49)	1.15 (1.01 to 1.31)	1.27 (1.12 to 1.44)

25(OH)D, 25-hydroxyvitamin D; CVD, cardiovascular disease

^a^Ordered by the baseline and median regression-derived 25(OH)D concentrations in the data (sex- and age-adjusted ranking, from highest to lowest: August, September, July, October, November, June, December, January, May, February, March, April). The ratio of the median 25(OH)D concentration was 1.00 (August, September; reference), 1.10 (July, October), 1.37 (June, November), 1.54 (January, December), 1.66 (February, May), and 1.79 (March, April)

^b^Coronary heart disease or stroke

^c^Estimated from Poisson regression models and adjusted for sex, attained age, and cohort

The shape of the association between seasonal changes in 25(OH)D and seasonal changes in CVD did not differ by low or high baseline 25(OH)D concentrations (*p* for interaction ≥ 0.10; S4 Fig). The comparison of trough and peak months of 25(OH)D was also similar in subgroup analyses by attained age, sex, and cohort (*p* for interaction ≥ 0.21; S5 Fig).

#### Association between 25(OH)D exposure and CVD outcomes.

Cox regression-derived HRs of CVD incidence and mortality by quarters and tenths of 25(OH)D status are shown in Table 4 and S6 Fig. In the age-, sex-, and season-adjusted model, the highest quarter of 25(OH)D had a strong inverse association with CVD incidence and mortality: the HR was reduced by 31 and 49%, respectively, compared to the lowest quarter (Table 4). In the multivariable-adjusted model, the inverse associations were largely attenuated but remained strong with HR reductions of 18% for CVD incidence and 36% for CVD mortality. HRs of secondary endpoints are presented in S9 Table. In brief, coronary heart disease, stroke, and heart failure had inverse associations with 25(OH)D status and atrial fibrillation had a null association with 25(OH)D status.

**Table 4:** HRs of CVD incidence and mortality, based on multiple imputed data and according to quarters of 25(OH)D status.

	Quarters of 25(OH)D concentration (nmol/L)^a^			
Endpoint	One (lowest)	Two	Three	Four (highest)
CVD incidence^b^				
No. of cases^c^	1570	1499	1296	1195
No. of person-years^c^	190,377	193,335	197,723	194,527
HR, model one (95% CI)^d^	1.00 (reference)	0.92 (0.85 to 0.98)	0.75 (0.70 to 0.81)	0.69 (0.64 to 0.75)
HR, model two (95% CI)^e^	1.00 (reference)	0.98 (0.91 to 1.05)	0.85 (0.79 to 0.92)	0.82 (0.76 to 0.89)
CVD mortality				
No. of cases^c^	876	757	613	488
No. of person-years^c^	230,630	235,295	238,691	235,082
HR, model one (95% CI)^d^	1.00 (reference)	0.82 (0.74 to 0.90)	0.64 (0.57 to 0.71)	0.51 (0.45 to 0.56)
HR, model two (95% CI)^e^	1.00 (reference)	0.89 (0.81 to 0.98)	0.75 (0.67 to 0.83)	0.64 (0.57 to 0.72)

25(OH)D, 25-hydroxyvitamin D; CVD, cardiovascular disease HR, hazard ratio

^a^Sex-, cohort- and calendar month-specific quarters. The ratio of the median 25(OH)D concentration was 1.00 (quarter one; reference) 1.50 (quarter two), 1.98 (quarter three), 2.86 (quarter four)

^b^Coronary heart disease or stroke

^c^The number of cases and person-years do not sum up to the entire study population in each analytical cohort, because of different exposure distributions across the imputed data sets (*n* = 20). Reported numbers are based on the participants who had complete data on 25(OH)D status

^d^Estimated from Cox regression models and adjusted for attained age, sex, cohort, and calendar week of blood sampling

^e^Estimated from Cox regression models and adjusted for attained age, sex, cohort, calendar week of blood sampling, body mass index, smoking, systolic blood pressure, use of antihypertensive drugs, total cholesterol, creatinine-estimated glomerular filtration rate, and history of CVD (i.e., coronary heart disease or stroke; only for mortality) and diabetes

The results did not markedly change in sensitivity analyses based on complete case data, irrespective of further adjustment for education, restriction of the follow-up time, or re-categorization of the exposure variable (S10 Table). There seemed to be a small degree of reverse causality in the association of 25(OH)D status with CVD mortality, especially when excluding prevalent cases of CVD, but not in the association with CVD incidence (relative differences in HR of 6.3 and 1.2%, respectively, in the models excluding the first three years of follow-up; S11 Table).

There was no evidence of an interaction between 25(OH)D status and attained age or sex in relation to CVD incidence (*p* for interaction ≥ 0.17; S7 and S8 Figs), with multivariable-adjusted HRs of 0.83 (95% CI: 0.75–0.92) and 0.80 (95% CI: 0.70–0.91) in men and women, respectively, for the comparison of extreme quarters. In contrast, there was evidence of an interaction by attained age for CVD mortality in the highest 25(OH)D category (*p* for interaction = 0.020 for quarter four compared to quarter one), with deviation from the time scale-fixed HR in participants above 80 years of age (S7 Fig). In the same quarter, CVD mortality also differed by sex (*p* for interaction = 0.019 compared to quarter one), with multivariable-adjusted HRs of 0.59 (95% CI: 0.51–0.68) in men and 0.76 (95% CI: 0.62–0.93) in women (S8 Fig). The exposure-endpoint associations by cohort are shown in S9 Fig, for which there was no clear evidence of between-cohort heterogeneity (*p* for interaction ≥ 0.052).

## Discussion

In this study, which included almost 80,000 participants from eight European cohorts, we have conducted detailed analyses on the association between seasonal changes in 25(OH)D and seasonal changes in CVD outcomes as well as on the association between individual 25(OH)D concentrations and CVD outcomes.

### Cross-sectional analyses

See S1 Text for a discussion of the cross-sectional findings.

### Prospective analyses

#### Seasonality of CVD outcomes and 25(OH)D status.

An often overlooked aspect of 25(OH)D in relation to health outcomes is the Earth’s axis deviation and angular rotation, which leads to a never-ending natural experiment with oscillating 25(OH)D concentrations on a season-to-season basis, at least in the Northern hemisphere. In 1981, it was hypothesized by Scragg that hypovitaminosis D was a major contributor to the observed winter excess in CVD outcomes, either via direct or indirect effects on thrombosis formation [26]. Since then, short-term reductions in 25(OH)D concentrations have in fact been associated with different measures of arterial stiffness and vascular dysfunction in observational data [34]. One feature that Scragg found especially attractive with his ecological hypothesis was that it was “testable”. When it finally was tested, some 34 years later in the SHHEC (*n* = 13,224), there was no evidence that seasonal variation in 25(OH)D had a large impact on seasonal variation in CVD outcomes [27]. Despite a 2:1 ratio of 25(OH)D concentrations between August and March, there was no peak or trough in CVD incidence, and the peak and trough in CVD mortality occurred near the solstices, preceding the extremes of 25(OH)D by at least two months. In addition, there was no increased outcome variability in persons with “low” 25(OH)D status, defined by below-median 25(OH)D concentrations; that is, persons who should experience more hypovitaminosis D during the winter. As such, Scragg’s hypothesis was rejected by the authors, given that any underlying mechanism explaining a seasonal variation in CVD outcomes must occur acutely (as acknowledged by Scragg himself [26]).

Our study was an extension of the original SHHEC paper, in which Scragg’s hypothesis was examined once again but with data from several geographical regions and with a larger number of participants, person-years, and CVD cases. As expected, there was a large difference in the median 25(OH)D concentration by calendar month, with the peak observed in August and the trough observed in March (1.79:1 ratio). The peak-to-trough ratio was also diluted by the Swedish cohort, which had the highest median 25(OH)D concentrations despite a non-full year (winter-to-spring) examination period (1.93:1 ratio, if that cohort had been excluded). Nonetheless, the trough months of 25(OH)D in March and April had a similar CVD incidence as the peak months of 25(OH)D in August and September. CVD mortality was slightly higher in trough months compared to peak months but not compared to other non-peak months of 25(OH)D, leading to a rather nonsensical trend across categories with increasing differences in median 25(OH)D concentrations. Importantly, the peak in CVD mortality in January preceded the trough in 25(OH)D by two to three months, not adhering to the cause-must-precede-outcome criterion of the Bradford Hill criteria [35]. The trough in CVD mortality also started in May, in adjacent to the trough in 25(OH)D. Finally, similar to the SHHEC study, there was no evidence of an increased susceptibility of CVD outcomes during the trough months of 25(OH)D in persons with “low” baseline 25(OH)D status, defined by a 25(OH)D concentration below the sex- and calendar month-specific median value in each cohort.

All in all, and based on the findings from our study and the previous SHHEC study, we feel comfortable to reject Scragg’s hypothesis that short-term reductions in 25(OH)D concentrations are a major contributor to seasonal increases in CVD outcomes. While a winter excess of CVD cannot be denied, more so for mortality than for incidence in our data, the underlying cause is likely multifactorial and not due to one single factor (e.g., temperature, air pollution, and respiratory infections) [24,25,36]. In analogy, we do believe that a lack of sunlight exposure during the winter plays a role, but to reduce that role to a mere 25(OH)D pathway is an oversimplification.

#### Association between 25(OH)D exposure and CVD outcomes.

The existing literature of observational studies on the association between 25(OH)D status and CVD outcomes is extremely large, to the point where it is almost hard to get an overview. Previous studies have reported an increased mortality of CVD [1–3], as well as an increased incidence of coronary heart disease [4], stroke [5], heart failure [6], and atrial fibrillation [7], in persons with low 25(OH)D concentrations. Similar findings have also been observed in persons with comorbid conditions like diabetes [37,38]. Our study does not add novelty to the overall body of observational data but confirms most of the previous findings, with rather strong but surprisingly similar associations of 25(OH)D status with mortality of CVD and with incidence of coronary heart disease, stroke, and heart failure. Except for an interaction by age and sex on the highest 25(OH)D quarter in the CVD mortality analysis, with a weaker association in old and female participants, the exposure-outcome associations were also consistent across multiple sensitivity and subgroup analyses. In contrast, we did not observe an association between 25(OH)D status and incidence of atrial fibrillation. In a meta-analysis by Liu et al, which included data from six studies (*n* = 5503 cases; *N* = 66,139 participants), vitamin D deficiency (< 50 nmol/L) was associated with an increased incidence of atrial fibrillation [7]. However, as discussed by Gaksch et al [3], most meta-analyses on 25(OH)D status and CVD outcomes have been hampered by between-study variations in the assay method of 25(OH)D, an issue to which the meta-analysis by Liu et al is no exception.

Whether the observational associations between 25(OH)D concentrations and CVD outcomes are causal have been debated for many years. The HRs in our study were largely attenuated by multivariable adjustment, notwithstanding that we could not adjust for a number of potential confounders (e.g., diet, alcohol, and physical activity). A causal role of 25(OH)D has not been supported by randomized clinical trials, in which vitamin D supplementation has neither reduced incidence and mortality of CVD [16–20,38] nor improved different biomarkers of glycaemia, inflammation, and lipids [39]. It should, however, be noted that vitamin D supplementation has been associated with an improved cardiac function in patients with heart failure (by reducing the left ventricular end-diastolic diameter and increasing the left ventricular ejection fraction) [40].

Up until recently, there was no evidence from Mendelian randomization studies of an association between genetically-predicted 25(OH)D concentrations and incidence or mortality of CVD [8–11]. However, three Mendelian randomization studies published in 2021 and 2022 (all based on the UK Biobank and utilizing a so-called non-linear randomization) did observe an inverse association with CVD incidence [12,13] and CVD mortality [12,14], especially in subgroup analyses of persons with low 25(OH)D concentrations. In addition, another Mendelian randomization study found an inverse association with heart failure incidence [15]. Since then, the methods and model assumptions of non-linear Mendelian randomization studies have been under great scrutiny [41]. Subsequently, one of the aforementioned studies was recently retracted and republished with an altered conclusion, no longer supporting a causal relationship between genetically-predicted 25(OH)D concentrations and CVD outcomes [12,42].

Despite methodological issues, the above-mentioned Mendelian randomization studies have re-ignited the debate on vitamin D supplementation and to whom such supplementation should be focused on. However, given that more than 40 years have passed since the publication of Scragg’s hypothesis, the scientific and medical community must at some point decide on the importance of 25(OH)D in the primary and secondary prevention of CVD.

### Strengths and limitations

The strengths of our study include the prospective design, the large sample size recruited from six European countries, and the use of harmonized data on 25(OH)D (i.e., analyzed in the same lab and with the same assay method). In addition, the MORGAM Data Centre has a long-standing expertise in data harmonization, leading to the best possible between-study alignment of outcomes and covariates. Finally, the detailed analysis of short-term changes in 25(OH)D in relation to seasonal variation in CVD adds novelty to the existing scientific literature.

Some limitations must be mentioned apart from the previously discussed possibility of unmeasured confounding. First, the 25(OH)D concentrations were estimated with a one-step immunoassay and not a high-performance liquid chromatography with tandem mass spectrometry [43]. In our own validation, the methods differed in magnitude of 25(OH)D concentrations but had a good correlation in terms of rank (S1 Text). Therefore, and since we did not use a Vitamin D Standardization Program protocol [44], the observed values of 25(OH)D could not be used to categorize participants as vitamin D sufficient, deficient, or insufficient (and we encourage others to be careful to use such categorization, especially if 25[OH]D is measured with an immunoassay). For the same reason, and to minimize the effect of within- and between-cohort differences in the calendar month of blood sampling, we did not analyze the exposure as a continuous variable in the Cox regression models. Second, there was a large difference in the pre-analytical storage time between cohorts (median time ranging from seven to 26 years). However, previous studies have shown that blood metabolites of 25(OH)D seem stable to storage duration [45] as well as to handling [46] and multiple freeze-thaw cycles [47]. Third, we only had a single 25(OH)D measurement for each participant (collected between 1984 and 2010), which, inevitably, led to some degree of exposure misclassification. The ranking of calendar months as peak or trough months of 25(OH)D in the Poisson regression models should be robust, considering the consistency of such ranking over time and in different countries, with the peaks in late summer to early autumn and the troughs in late winter to early spring [22,23]. In contrast, the ranking of individual 25(OH)D concentrations is more vulnerable to misclassification at baseline and during follow-up, mainly affecting the percentile-based categorization in the Cox regression models. It should, however, be noted that a single 25(OH)D measurement has been shown to have a similar long-term tracking as many other CVD risk factors [27,48], with correlation coefficients of 0.61 at a five-year interval [49], 0.57 at a six-year interval [50], and 0.52 at a 14-year interval [48]. In our study, there was also no evidence of between-cohort heterogeneity, despite large differences in the length of follow-up (S1 Table). Fourth, we lacked information on vitamin D supplements and whether its use varied over time and by calendar season. Fifth, even though the validation of CVD endpoints was systematic and detailed, and mainly based on medical reviews or hospital and mortality registers, some endpoint misclassification is likely to have occurred. Finally, our study only included individuals from European countries and its generalisability to other regions is unknown.

## Conclusion

Our study adds important perspectives to the existing literature on the association between 25(OH)D status and CVD outcomes, providing no support for the long-standing hypothesis that the winter excess in CVD is due to short-term reductions in 25(OH)D. The CVD-25(OH)D seasonal hypothesis seems overdue for correction but needs to be tested in other studies, preferably with repeated measurements of 25(OH)D and among non-Europeans. With respect to individual 25(OH)D concentrations, we confirmed most of the findings from previous observational studies, with increased CVD incidence and mortality in persons with low 25(OH)D concentrations. Whether these are causal associations are questionable given the current evidence from randomized clinical trials and Mendelian randomization studies.

## Supporting information

S1 Checklist Strengthening the Reporting of Observational Studies in Epidemiology (STROBE) statement. (PDF)

S2 Checklist Inclusivity in global research. (PDF)

S1 Text Supporting Methods and Discussion. (PDF)

S1 Table Overview and description of the included cohorts. (PDF)

S2 Table Baseline and follow-up data on CVD endpoints by cohort. (PDF)

S3 Table Missing data (%) by CVD endpoint and analytical model. (PDF)

S4 Table Baseline characteristics of study participants by cohort. (PDF)

S5 Table Cohort-specific rates of CVD incidence and mortality per 10,000 person-years in participants aged 60–69 years and according to sex and decade. The numbers within parenthesis (in smaller font) refer to the total number of cases in each strata.
(PDF)

S6 Table Co-occurrence of incident CVD endpoints during follow-up. (PDF)

S7 Table Rate ratios of CVD incidence and mortality by three-, four-, and six-month calendar periods. (PDF)

S8 Table Incidence rate ratios of coronary heart disease and stroke by two-month calendar periods (ordered by 25[OH]D status). (PDF)

S9 Table HRs of secondary CVD endpoints, based on complete case data and according to quarters of 25(OH)D status. (PDF)

S10 Table Sensitivity analyses of the association of 25(OH)D status with CVD incidence and mortality using (i) complete case data, (ii) complete case data with adjustment for education, (iii) complete case data with restriction of the follow-up time, and (iv) complete case data with re-categorization of the exposure. (PDF)

S11 Table Examination of reverse causality in the association of 25(OH)D status with CVD incidence and mortality. (PDF)

S1 Fig Seasonal variation of 25-hydroxyvitamin D (25[OH]D) status by age (first row), sex (second row), decade (third row), and country (fourth row). Data from Sweden and Finland are not included in the country-specific analysis due to a non-full year examination period (winter-to-spring); however, full season data from Sweden and Finland are available elsewhere (Klingberg et al, *Endocrine* 49, 800–808; Stridh et al, *Sci Rep* 11, 20989).
(PDF)

S2 Fig Quantile regression-derived median difference in 25-hydroxyvitamin D (25[OH]D) status by age (modelled using four-knot restricted cubic splines at the fifth, 35th, 65th, and 95th percentile). The solid lines represent the differences in median 25(OH)D concentrations and the dashed lines represent the 95% CI. Estimates were adjusted for sex and season of sampling (winter, spring, summer, and fall). The reference value was set to the median value of age in each cohort.
(PDF)

S3 Fig Quantile regression-derived median difference in 25-hydroxyvitamin D (25[OH]D) status by kidney function (according to creatinine-estimated glomerular filtration rate [eGFR]; modelled using four-knot restricted cubic splines at the fifth, 35th, 65th, and 95th percentile). The solid line represents the difference in median 25(OH)D concentrations and the shaded area represents the 95% CI (on the left y-axis). The histogram represents the distribution of eGFR in the study population (on the right y-axis). Estimates were adjusted for sex, age (continuous using four-knot restricted cubic splines, years), and season of sampling (winter, spring, summer, and fall). The reference value was set to the median value of eGFR in the study population (85 ml/min/1.73 m^2^).
(PDF)

S4 Fig Rate ratios of cardiovascular disease incidence and mortality by two-month calendar periods (ordered by 25-hydroxyvitamin D [25(OH)D] status) and according to low or high 25(OH)D status at the start of follow-up (using the sex-, calendar month-, and cohort-specific median value as the cut-off value). Circles and spikes represent point estimates and 95% CI, which were derived from Poisson regression models and adjusted for sex, attained age, and cohort. The reported *p* values for interaction were calculated by including an interaction term between the two-month calendar periods and 25(OH)D status in the Poisson regression model and testing its coefficients equal to zero. The p value for an overall interaction by testing the coefficients jointly equal to zero was 0.98 and 0.67, respectively, for cardiovascular disease incidence and mortality.
(PDF)

S5 Fig Rate ratios of cardiovascular disease incidence and mortality for the trough (March, April) compared to the peak (August, September) months of 25-hydroxyvitamin D status and according to attained age, sex, and cohort. Circles and capped spikes represent point estimates and 95% CI, which were derived from Poisson regression models and adjusted for sex, attained age, and cohort (where appropriate). The *p* values refer to interaction effects (see S1 Text for details on how the tests for interaction were conducted).
(PDF)

S6 Fig Hazard ratios of cardiovascular disease incidence and mortality by tenths of 25-hydroxyvitamin D (25[OH]D) status. Circles and spikes represent point estimates and 95% CI, which were based on multiple imputed data, derived from Cox regression models, and adjusted for the same covariates as in Table 4.
(PDF)

S7 Fig Time-varying hazard ratios of cardiovascular disease incidence (page A) and mortality (page B) for the second to fourth quarter of 25-hydroxyvitamin D (25[OH]D) status compared to the first quarter, according to attained age during follow-up (time scale). The solid and long dashed lines represent time-varying estimates and 95% CI, which were based on complete case data and derived from a Cox regression model that included an interaction term between 25(OH)D status (in quarters) and attained age (as a continuous variable, modeled using four-knots restricted cubic splines). The short dashed lines represent the time-fixed estimate based on complete case data. All estimates were adjusted for the same covariates as in Table 4. The reported *p* values for interaction were calculated by testing the second and third spline transformation jointly equal to zero.
(PDF)

S8 Fig Hazard ratios of cardiovascular disease incidence and mortality by quarters of 25-hydroxyvitamin D (25[OH]D) status and according to sex. Circles and spikes represent point estimates and 95% CI, which were based on multiple imputed data, derived from Cox regression models, and adjusted for the same covariates as in Table 4. The reported *p* values for interaction were calculated by including an interaction tern between 25(OH)D status and sex in the Cox regression model and testing its coefficients equal to zero. The *p* value for an overall interaction (testing the coefficients jointly equal to zero) was 0.68 and 0.10, respectively, for cardiovascular disease incidence and mortality.
(PDF)

S9 Fig Cohort-specific case distribution of cardiovascular disease incidence (page A) and mortality (page B) as well as hazard ratios (HRs) and 95% CI for the second to fourth quarter of 25-hydroxyvitamin D status compared to the first quarter. HRs and 95% CI were based on multiple imputed data and derived from Cox regression models, which were adjusted for the same covariates as in Table 4. The dashed lines represent the point estimates in the pooled analysis. The reported *p* values for interaction were calculated using the Cochran Q test from a random-effects meta-analysis.
(PDF)
