# Supplementary material for: Cardiovascular disease outcomes in relation to 25-hydroxyvitamin D and its seasonal variation: Results from the BiomarCaRE consortium
Source: PLoS One. 2025 Apr 24;20(4):e0319607. doi: 10.1371/journal.pone.0319607 (PMC12021148; doi:10.1371/journal.pone.0319607)
Supplement: S1 Checklist — (PDF) [file pone.0319607.s001.pdf]

STROBE Statement—Checklist of items that should be included in reports of ***cohort studies***

	Item No	Recommendation
**Title and abstract**<br>**Included on page 1 and 3**	1	(*a*) Indicate the study's design with a commonly used term in the title or the abstract
		(*b*) Provide in the abstract an informative and balanced summary of what was done and what was found
**Introduction**		
Background/rationale<br>**Included on page 4**	2	Explain the scientific background and rationale for the investigation being reported
Objectives<br>**Included on page 5**	3	State specific objectives, including any prespecified hypotheses
**Methods**		
Study design<br>**Included on page 5, 6, and 7**	4	Present key elements of study design early in the paper
Setting<br>**Included on page 5, 6, and 7; in Fig 1; and in S1 Table**	5	Describe the setting, locations, and relevant dates, including periods of recruitment, exposure, follow-up, and data collection
Participants<br>**Included on page 5; in Fig 1; and in S1 Table**	6	(*a*) Give the eligibility criteria, and the sources and methods of selection of participants. Describe methods of follow-up
		(*b*) For matched studies, give matching criteria and number of exposed and unexposed
Variables<br>**Included on page 5, 6, 7, 8, 9, and 10; in S1 Text; and in S2 Table**	7	Clearly define all outcomes, exposures, predictors, potential confounders, and effect modifiers. Give diagnostic criteria, if applicable
Data sources/ measurement<br>**Included on page 5, 6, 7, 8, 9, and 10; in S1 Text; and in S2 Table**	8*	For each variable of interest, give sources of data and details of methods of assessment (measurement). Describe comparability of assessment methods if there is more than one group
Bias<br>**Included on page 5, 6, 7, 8, 9, and 10; in S1 Text; and in S1 Table**	9	Describe any efforts to address potential sources of bias
Study size<br>**Included on page 6, 7, 8, 9, and 10; in Fig 1; and in Table 2**	10	Explain how the study size was arrived at
Quantitative variables<br>**Included on page 8, 9, and 10; and in S1 Text**	11	Explain how quantitative variables were handled in the analyses. If applicable, describe which groupings were chosen and why
Statistical methods<br>**Included on page 7, 8, 9, and 10; and in S1 Text**	12	(*a*) Describe all statistical methods, including those used to control for confounding
		(*b*) Describe any methods used to examine subgroups and interactions
		(*c*) Explain how missing data were addressed
		(*d*) If applicable, explain how loss to follow-up was addressed
		(*e*) Describe any sensitivity analyses
**Results**		
Participants	13*	(a) Report numbers of individuals at each stage of study—eg numbers

**Included on page 8, 11, and 14; in Fig 1; and in Table 2**		potentially eligible, examined for eligibility, confirmed eligible, included in the study, completing follow-up, and analysed
		(b) Give reasons for non-participation at each stage
		(c) Consider use of a flow diagram
Descriptive data<br>**Included on page 11; in Table 1; in S3 and S4 Table; and in S1 and S2 Fig**	14*	(a) Give characteristics of study participants (eg demographic, clinical, social) and information on exposures and potential confounders
		(b) Indicate number of participants with missing data for each variable of interest
		(c) Summarise follow-up time (eg, average and total amount)
Outcome data<br>**Included on page 14; in Table 2; and in S5 and S6 Table**	15*	Report numbers of outcome events or summary measures over time
Main results<br>**Included on page 16 and 18; in Table 3 and 4; and in Fig 2**	16	(*a*) Give unadjusted estimates and, if applicable, confounder-adjusted estimates and their precision (eg, 95% confidence interval). Make clear which confounders were adjusted for and why they were included
		(*b*) Report category boundaries when continuous variables were categorized
		(*c*) If relevant, consider translating estimates of relative risk into absolute risk for a meaningful time period
Other analyses<br>**Included on page 16, 18, and 20; in S7 to S11 Tables; and in S3 to S8 Fig**	17	Report other analyses done—eg analyses of subgroups and interactions, and sensitivity analyses
**Discussion**		
Key results<br>**Included on page 21, 22, 23, 24, 25, and 26**	18	Summarise key results with reference to study objectives
Limitations<br>**Included on page 25 and 26**	19	Discuss limitations of the study, taking into account sources of potential bias or imprecision. Discuss both direction and magnitude of any potential bias
Interpretation<br>**Included on page 22, 23, 24, and 26**	20	Give a cautious overall interpretation of results considering objectives, limitations, multiplicity of analyses, results from similar studies, and other relevant evidence
Generalisability<br>**Included on page 26**	21	Discuss the generalisability (external validity) of the study results
**Other information**		
Funding<br>**Included in the financial disclosure section of the submission system**	22	Give the source of funding and the role of the funders for the present study and, if applicable, for the original study on which the present article is based

*Give information separately for exposed and unexposed groups.

Note: An Explanation and Elaboration article discusses each checklist item and gives methodological background and published examples of transparent reporting. The STROBE checklist is best used in conjunction with this article (freely available on the Web sites of PLoS Medicine at http://www.plosmedicine.org/, Annals of Internal Medicine at http://www.annals.org/, and Epidemiology at http://www.epidem.com/). Information on the STROBE Initiative is available at http://www.strobe-statement.org.
