# Supplementary material for: Cardiovascular disease outcomes in relation to 25-hydroxyvitamin D and its seasonal variation: Results from the BiomarCaRE consortium
Source: PLoS One. 2025 Apr 24;20(4):e0319607. doi: 10.1371/journal.pone.0319607 (PMC12021148; doi:10.1371/journal.pone.0319607)
Supplement: S1 Text — (PDF) [file pone.0319607.s003.pdf]

Supporting Material and Methods

Details of the 25-hydroxyvitamin D (25[OH]D) measurement

Serum or plasma analyses of 25(OH)D were conducted between 2009 and 2018 at the Biomarkers for Cardiovascular Risk Assessment in Europe (BiomarCaRE) laboratory in Germany (located in Mainz up until 2012, thereafter in Hamburg) using a one-step immunoassay on the Abbott ARCHITECT i2000 (Abbott Diagnostics, Abbott Park, IL, USA; analytical range 8.5 to 399.4 nmol/L) [Cavalier et al, *Clin Chem Lab Med* 55, 378–384]. Previous research has shown that serum and plasma measurements of 25(OH)D are highly similar [Colak et al, *Biochem Med (Zagreb)* 23, 321–325]. No evidence of laboratory drift was observed during the study period. The 25(OH)D concentrations are reported in nmol/L, which can be converted to ng/mL by dividing with a factor of 2.496.

In our own validation study, using a subsample of the Monitoring of Trends and Determinants in Cardiovascular disease (MONICA) Northern Sweden, the immunoassay had a good correlation in terms of rank (Spearman's coefficient [*r*] = 0.82 to 0.91, irrespective of sex and age group)—but underestimated the 25(OH)D concentrations—compared to a high-performance liquid chromatography with tandem mass spectrometry (HPLC-MS/MS; traceable to the National Institute of Standards and Technology's Standard Reference Material 972) [Summerhays et al*, Eur J Nutr* 59, 3037–3044]. A similar underestimation of 25(OH)D concentrations by the Abbott ARCHITECT i2000 has been reported in other studies [Wyness & Straseski, *Clin Biochem* 48, 1089–1096].

The cohort-specific years of blood sampling, years of 25(OH)D analysis, and intra-assay and inter-assay coefficients of variation (CV) were as follows:

MONICA Northern Sweden
Year of blood sampling: 1986 to 2009
Year of 25(OH)D analysis: 2016 to 2018
Intra- and inter-assay CV: 1.1 to 3.0 and 4.3 to 9.1

FINRISK 1997
Year of blood sampling: 1997
Year of 25(OH)D analysis: 2012 to 2013
Intra- and inter-assay CV: 2.8 to 4.5 and 6.8 to 7.2

Scottish Heart Health Extended Cohort (SHHEC)
Year of blood sampling: 1984 to 1995
Year of 25(OH)D analysis: 2009 to 2012
Intra- and inter-assay CV: 5.0 to 9.5 and 1.4 to 8.7

MONICA/Cooperative Health Research in the Region of Augsburg (KORA)
Year of blood sampling: 1994 to 2001
Year of 25(OH)D analysis: 2015
Intra- and inter-assay CV: 2.7 to 3.5 and 3.7 to 4.2

MONICA Brianza

Year of blood sampling: 1986 to 1993

Year of 25(OH)D analysis: 2015

Intra- and inter-assay CV: 4.5 and 2.9 to 6.8

Moli-sani

Year of blood sampling: 2005 to 2010

Year of 25(OH)D analysis: 2014 to 2015

Intra- and inter-assay CV: 2.5 to 4.5 and 5.2 to 8.0

Malattie Aterosclerotiche Istituto Superiore di Sanità (MATISS)

Year of blood sampling: 1993 to 1996

Year of 25(OH)D analysis: 2016

Intra- and inter-assay CV: 2.8 to 2.9 and 4.0 to 5.6

MONICA-Catalonia

Year of blood sampling: 1986 to 1992

Year of 25(OH)D analysis: 2016

Intra- and inter-assay CV: 2.8 and 3.5 to 8.4

Equation for the estimation of the glomerular filtration rate

Creatinine-estimated glomerular filtration rate (crea-eGFR) was calculated using the following equation from the Chronic Kidney Disease Epidemiology (CKD-EPI) Collaboration (Levey et al, *Ann Intern Med,* 150, 604–612):

crea-eGFR (ml/min/1.73 m^2) = 141 x $\min(S_{cr}/\kappa, 1)^{\alpha}$ x $\max(S_{cr}/\kappa, 1)^{-1.209}$ x 0.993^{age} x 1.018[if female], where:

S_{cr} is serum creatinine in mg/dL

κ is 0.7 for females and 0.9 for males

α is −0.329 for females and −0.411 for males

min is the minimum of S_{cr}/κ or 1

max is the maximum of S_{cr}/κ or 1

Additional details of the statistical analyses

Poisson regression analyses: To calculate monthly incidence and mortality rates, we used time since study entry (in days) as the time scale and split each observation on the last day of each calendar month throughout the entire follow-up period (creating up to 12 million observations, depending on the endpoint). The time scale was then altered to attained age (in years), after which each observation was further split by ten-year age groups (<30 years, 30 to 39 years, 40 to 49 years, etc.; up to >100 years). Data were then collapsed by calendar month, sex, age group, and cohort. (Nota bene: No calculations were made for heart failure or atrial fibrillation to avoid low-detection bias

during the summer—that is, a time period when the health care system is often less productive because of staff vacation—especially since these diseases are in general less acute than coronary heart disease and stroke.)

Evaluation of the proportional hazards assumption: To examine the assumption of time-fixed hazard ratios (i.e., proportional hazards), the Cox regression model included an interaction term between 25(OH)D status (in quarters) and attained age (as a continuous variable, split at 0.5 year intervals, and modeled using four-knots restricted cubic splines). A formal test for non-proportional hazards (= an interaction by attained age) was carried out via the Wald test, testing the second and third spline transformation jointly equal to zero. This analysis was, because of computational reasons, conducted using complete case data.

Multiple imputation: The mi impute [chained] command in Stata were used to impute the data. A total of 20 data sets were created and combined using Rubin's rule [White et al, Stat Med 30, 377–399]. The regular variables were age, sex, cohort, calendar year of examination, calendar month of examination, calendar week of examination, endpoint status, and the Nelson–Aalen estimate of the cumulative hazard function. The imputed variables were filled in using predictive mean matching (i.e., systolic blood pressure, 25(OH)D, total cholesterol, and creatinine-estimated glomerular filtration rate) or logistic regression (i.e., smoking, antihypertensive medication, history of cardiovascular disease [CVD], and history of diabetes). Using linear regression instead of predictive mean matching for the continuous variables had negligible influence on the results. Categorization of 25(OH)D, as well as spline transformations of the other continuous variables, was conducted after the imputation (creating so-called passive variables).

Re-categorization of the exposure variable: To account for long baseline examination periods, the cohorts were further categorized according to calendar year of examination as follows: MONICA Northern Sweden (3 levels: 1986–1990, 1994–1999, and 2004–2009), FINRISK 1997 (no further categorization), SHHEC (2 levels: 1984–1989 and 1992–1995), MONICA/KORA (2 levels: 1994–1995 and 1999–2001), MONICA Brianza (2 levels: 1986–1989 and 1990–1994), Moli-sani (no further categorization), MATISS (no further categorization), and MONICA-Catalonia (2 levels: 1986–1988 and 1990–1992). The quarters of 25(OH)D status were then re-categorized to be sex-, subcohort-, and calendar month-specific; and the multivariable model was adjusted for the subcohort variable (categorical, 14 levels).

Models used to examine reverse causality bias: (1) Delaying the start of follow-up with one, two, and three years; (2) further excluding participants with prevalent CVD at baseline (only applied in the mortality analysis, since the prevalent cases were already excluded in the incidence analysis); and (3) further excluding participants with N-terminal pro-B-type natriuretic peptide or high-sensitive troponin I concentrations above the 99th percentile in the disease-free population at baseline (i.e., concentrations that might indicate undiagnosed CVD).

Tests for interaction: To test for an interaction by 25(OH)D status (categorical, two levels) on the association of two-month calendar periods (categorical, six levels) with CVD incidence and mortality,

we included an interaction term between the two variables in the Poisson regression model. The Wald test was then used to obtain *p* values for interaction (by testing the coefficients of the interaction term equal to zero). The same methodology was used to test for interactions by attained age (categorical, two levels) and sex (categorical, two levels) in the Poisson regression model as well as to test for an interaction by sex (categorical, two levels) on the association of 25(OH)D status (categorical, quarters) in the Cox regression model. To test for an interaction by cohort (categorical, eight levels) in the Poisson regression model (on the association of two-month calendar periods [categorical, six levels]) and Cox regression model (on the association of 25[OH]D status [categorical, quarters]), we performed two random-effects meta-analyses of the cohort-specific estimates. The Cochran Q test was then used to obtain *p* values for interaction. (Nota bene: For details on the interaction analysis by attained age [time scale] on the association of 25[OH]D status [categorical, quarters] with CVD incidence and mortality, see subheading ”Evaluation of the proportional hazards assumption” above.)

Supporting Discussion

Cross-sectional findings

In a previous study from the BiomarCaRE project, we observed a north-to-south gradient of 25(OH)D status, with higher concentrations in the northernmost countries (i.e., Sweden and Finland) compared to the southernmost countries (i.e., Spain and Italy) [Oskarsson et al, *Br J Nutr* 128, 2208–2218]. While this might seem counterintuitive, given that 25(OH)D concentrations are largely affected by sunlight exposure [Holick, *Am J Clin Nutr* 79, 362–371], the same finding has been observed in many other studies conducted during the 1980s [van der Wielen et al, *Lancet* 346, 207–210], 1990s [Zheng et al, *J Clin Endocrinol Metab* 104, 1293–1303], and 2000s [Deleskog et al, *Arterioscler Thromb Vasc Biol* 33, 2633–2638]. In the current study, the Swedish and Finnish cohorts differed from the other cohorts by having an opposite association between age and 25(OH)D status (i.e., positive instead of inverse). The Swedish cohort also differed from the other cohorts by having an opposite association between female sex and 25(OH)D status (i.e., positive instead of inverse). While the reasons for these observations are unclear, it could be explained by between-country differences in consumption of vitamin D-rich foods [Jenab et al, *Eur J Clin Nutr* 63, Suppl. 4, S150–S178], food fortification policies [Oskarsson et al, *Br J Nutr* 128, 2208–2218], and use of vitamin D supplements [Skeie et al, *Eur J Clin Nutr* 63, Suppl. 4, S226–S238]. Apart from age and sex, the cross-sectional associations were rather homogenous with lower 25(OH)D concentrations for CVD risk factors (e.g., obesity, smoking, and low education) and prevalent diseases (e.g., CVD, hypertension, and diabetes). One interesting finding was that higher 25(OH)D concentrations were associated with a worse kidney function (as measured by creatinine-estimated glomerular filtration rate). Although not without controversy in the light of previous research [Agarwal, *Clin J Am Soc Nephrol* 4, 1523–1528], it should be noted that the same finding has been observed in a recent Mendelian randomization study [Teumer et al, *Nephrol Dial Transplant* 33, 2139–2145] and in a recent analysis of the National Health and Nutrition Examination Survey [Geng et al, *Front Nutr* 8, 716878]. The underlying mechanism of this observation is unknown and requires future research.
