# Supplementary material for: Cardiovascular disease outcomes in relation to 25-hydroxyvitamin D and its seasonal variation: Results from the BiomarCaRE consortium
Source: PLoS One. 2025 Apr 24;20(4):e0319607. doi: 10.1371/journal.pone.0319607 (PMC12021148; doi:10.1371/journal.pone.0319607)
Supplement: S1 Table — (PDF) [file pone.0319607.s004.pdf]

Cohort	Country	Description
MONICA Northern Sweden	Sweden	The Northern Sweden MONICA study consists of seven population-based surveys (in 1986, 1990, 1994, 1999, 2004, and 2009 as well as 2014 [not included in the current study]) in the two northernmost counties of Sweden (i.e., Norrbotten and Västerbotten). Individuals were randomly selected from population registers and stratified for age (25 to 64 years in 1986 and 1990; 25 to 74 years in 1994 to 2014) and sex. Overall, the participation rate ranged from 63 to 81%. Endpoints (i.e., coronary heart disease, stroke, heart failure, atrial fibrillation, and mortality) were identified via linkage to the National Patient Register and the National Causes of Death Register. The follow-up period ended on December 31, 2011. All surveys were approved by the Regional Ethical Committee at Umeå University.<br>*Details: www.thl.fi/publications/morgam/cohorts/full/sweden/swe-nswa.htm*
FINRISK 1997	Finland	The FINRISK 1997 study is a population-based survey that was carried out in five districts of Finland, including North Karelia, northern Savo (former Kuopio), southwestern Finland, Oulu province, and the region of Helsinki and Vantaa. A stratified random sample of men and women (aged 25 to 74 years) was drawn from the National Population Register. The participation rate was 73%. The National Hospital Discharge Register, the National Causes of Death Register, and the National Drug Reimbursement Register were used to identify endpoints (i.e., coronary heart disease, stroke, heart failure, atrial fibrillation, and mortality). The follow-up period ended on December 31, 2010. The study was approved by the Ethical Committee of the National Public Health Institute.<br>*Details: www.thl.fi/publications/morgam/cohorts/full/finland/fin-fina.htm*
SHHEC	Scotland	The SHHEC consists of two overlapping studies: the Scottish Heart Health Study, which randomly recruited men and women (aged 40 to 59 years) across 22 Scottish districts in 1984 to 1987; and the Scottish MONICA study, which similarly recruited men and women (aged 25 to 64 years) in Edinburgh (in 1986) and Glasgow (in 1986, 1989, 1992 [up to 75 years of age], and 1995) as part of the WHO MONICA Project. Overall, the participation rate ranged from 59 to 74%. The Scottish National Health Service Central Register and the Scottish Record Linkage System were used to identify endpoints (i.e., coronary heart disease, stroke, heart failure, atrial fibrillation, and mortality). The follow-up period ended on December 31, 2009. The Scottish Heart Health Study and the Scottish MONICA study were approved by the then Privacy Advisory Committee and Chief Scientist Committee of the Scottish Home and Health Department. They were later approved by approximately 30 individual local research ethics committees.<br>*Details: www.thl.fi/publications/morgam/cohorts/full/uk/unk-sco.htm*

MONICA/KORA	Germany	The MONICA/KORA cohort consists of representative surveys from the city of Augsburg and the regions of Landkreis Augsburg and Landkreis Aichach-Friedberg in southern Germany. Municipality lists and population registers were used as sampling frames for the first and second stage of a two-stage random sampling procedure. The second stage of the sampling was stratified by sex and age. The baseline examinations in 1994 and 1995 (subcohort 3; part of the WHO MONICA project) and in 1999 to 2001 (subcohort 4; KORA) consisted of participants aged 25 to 74 years, with participation rates of 74 and 72%, respectively. Endpoints (i.e., coronary heart disease, stroke, and mortality) were identified through the MONICA/KORA Augsburg coronary event registry, follow-up questionnaires (in 2002 and 2009), population registers, and death certificates. The follow-up period ended on December 31, 2009. The studies were approved by the local authorities and conducted in accordance with the data protection regulations. The KORA study was also approved by the Ethics Committee of the Bavarian Chamber of Physicians.<br>*Details: www.thl.fi/publications/morgam/cohorts/full/germany/ger-auga.htm*
MONICA Brianza	Italy	The MONICA Brianza cohort is a prospective study of individuals in Brianza (aged 25 to 64 years), an area located between Milan and the Swiss border in northern Italy. Samples were stratified for sex and age and randomly drawn in 1986, 1990, and 1993. Overall, the participation rate ranged from 67 to 69%. Each municipality was contacted in the beginning of 1999, 2004, and 2011 for information about emigration from the municipality, present residency, and vital status. Endpoints (i.e., coronary heart disease, stroke, and mortality) were identified via hospital discharge records and death certificates. The follow-up period ended on December 31, 2008. The cohort was approved by the Comitato Etico Azienda Ospedaliera San Gerardo-Monza.<br>*Details: https://www.thl.fi/publications/morgam/cohorts/full/italy/ita-bria.htm*
Moli-sani	Italy	The Moli-sani cohort was established in the Molise region in southern Italy from city hall registries by a multistage sampling procedure. First, townships were sampled in major areas by cluster sampling; then, within each township, participants aged 35 years or older were selected by simple random sampling. The samples were drawn from 2005 to 2010 and the overall participation rate was 70%. Mortality and hospital discharge registries were used to identify endpoints (i.e., coronary heart disease, stroke, heart failure, atrial fibrillation, and mortality). The follow-up period ended on December 31, 2011 (coronary heart disease) and December 31, 2015 (mortality, stroke, atrial fibrillation, and heart failure). The study was approved by the Rome Catholic University Ethical Committee.<br>*Details: https://www.thl.fi/publications/morgam/cohorts/full/italy/ita-mola.htm*

MATISS	Italy	The MATISS study started in 1984 as a project on non-communicable diseases in central Italy. Four municipalities were involved: three receiving community treatment and one acting as control. Baseline (1984) and four-year follow-up examinations were used to evaluate, in a random sample of the general population, the community treatment areas compared to the control area. In 1993 to 1996, the cohorts were re-examined and a new random sample (which was used as the baseline for the current study), stratified by age (20 to 79 years) and sex, was enrolled from the residence registry (participation rate 60%). From the first screening to the last screening, municipalities were contacted every five years for information about vital status, emigration, and residency; from 1996 onwards, municipalities were contacted every year. Endpoints (i.e., coronary heart disease, stroke, and mortality) were identified via linkage to mortality and hospital discharge registries or via death certificates and re-examinations. The follow-up period ended on December 31, 2004. The MATISS study was approved by the Ethical Committee of the Istituto Superiore di Sanità-ISS.<br>*Details: https://www.thl.fi/publications/morgam/cohorts/full/italy/ita-roma.htm*
MONICA-Catalonia	Spain	The MONICA-Catalonia cohort consists of two population-based surveys from the central area of Catalonia and parts of the metropolitan area of Barcelona in northeastern Spain. The first stage of sampling drew a random sample from nine municipalities with a probability proportional to the population size. In the second stage, age- and sex-stratified random samples of individuals aged 25 to 64 years were drawn from the municipal population registries. Baseline examinations were carried out in 1986 to 1988 (cohort 1; participation rate 74%) and in 1990 to 1992 (cohort 2; participation rate 67%). Endpoints in cohort 1 (i.e., coronary heart disease, stroke, heart failure, atrial fibrillation, and mortality) and cohort 2 (i.e., coronary heart disease, stroke, and mortality) were assessed through follow-up questionnaires and record linkage with MONICA registers, national and regional mortality index registers, and hospital discharge registers. The follow-up period ended on May 31, 1994 (heart failure in cohort 1), December 31, 1997 (all other endpoints in cohort 1), and April 30, 1999 (cohort 2). The project was approved by the Board of the former Institute of Health Studies, Department of Health and Social Security, Generalitat of Catalunya.<br>*Details: https://www.thl.fi/publications/morgam/cohorts/full/spain/spa-cata.htm*

KORA, Cooperative Health Research in the Region of Augsburg; MATISS, Malattie Aterosclerotiche Istituto Superiore di Sanità; MONICA, Monitoring of Trends and Determinants in Cardiovascular disease; SHHEC, Scottish Heart Health Extended Cohort; WHO, World Health Organization
