# Supplementary material for: Cardiovascular disease outcomes in relation to 25-hydroxyvitamin D and its seasonal variation: Results from the BiomarCaRE consortium
Source: PLoS One. 2025 Apr 24;20(4):e0319607. doi: 10.1371/journal.pone.0319607 (PMC12021148; doi:10.1371/journal.pone.0319607)
Supplement: S2 Table — (PDF) [file pone.0319607.s005.pdf]

CVD endpoint	Cohort[a] MONICA Northern Sweden	FINRISK 1997	SHHEC	MONICA/ KORA	MONICA Brianza	Moli-sani	MATISS	MONICA-Catalonia[b]
Coronary heart disease								
Baseline	Yes	Yes	Yes	Yes	Yes	Yes	Yes	Yes
Follow-up[c]	Yes	Yes	Yes	Yes	Yes	Yes	Yes	Yes
Stroke								
Baseline	Yes	Yes	Yes	Yes	Yes	Yes	Yes	Yes
Follow-up	Yes	Yes	Yes	Yes	Yes	Yes	Yes	Yes
Heart failure								
Baseline	Yes	Yes	Yes	Yes	No	Yes	No	Yes
Follow-up	Yes	Yes	Yes	No	No	Yes	No	Yes
Atrial fibrillation								
Baseline	Yes	Yes	Yes	No	No	Yes	No	Yes
Follow-up	Yes	Yes	Yes	No	No	Yes	No	Yes
CVD mortality								
Follow-up[d]	Yes	Yes	Yes	Yes	Yes	Yes	Yes	Yes

CVD, cardiovascular disease; KORA, Cooperative Health Research in the Region of Augsburg; MATISS, Malattie Aterosclerotiche Istituto Superiore di Sanità; MONICA, Monitoring of Trends and Determinants in Cardiovascular disease; SHHEC, Scottish Heart Health Extended Cohort

[a] Details of the diagnostic procedures in each cohort are available from https://www.thl.fi/publications/morgam/cohorts/full/contents.htm

[b] With respect to heart failure, MONICA-Catalonia had baseline data in both cohorts and follow-up data in cohort 1 (but without exact dates of diagnosis). With respect to atrial fibrillation, MONICA-Catalonia had baseline and follow-up data in cohort 1 (including exact dates of diagnosis)

[c] Myocardial infarction (all cohorts), unstable angina pectoris (all cohorts except MONICA/KORA, MONICA Brianza, and MATISS), cardiac revascularization (all cohorts except MONICA/KORA), and unclassifiable death (all cohorts)

[d] Coronary death, stroke death, or unclassifiable death
