# Supplementary material for: Cardiovascular disease outcomes in relation to 25-hydroxyvitamin D and its seasonal variation: Results from the BiomarCaRE consortium
Source: PLoS One. 2025 Apr 24;20(4):e0319607. doi: 10.1371/journal.pone.0319607 (PMC12021148; doi:10.1371/journal.pone.0319607)
Supplement: S3 Table — (PDF) [file pone.0319607.s006.pdf]

Analytical model	CVD endpoint CHD (*n* = 76,434)	Stroke (*n* = 77,204)	HF[a] (*n* = 56,888)	AF[b] (*n* = 58,975)	CVD mortality (*n* = 79,570)
Multivariable model one[c]	6.8	6.9	7.7	7.7	7.6
Body mass index	0.3	0.3	0.2	0.2	0.3
Smoking	<0.1	<0.1	<0.1	<0.1	0.4
Systolic blood pressure	0.1	<0.1	<0.1	<0.1	0.1
Antihypertensives use	0.6	0.6	0.8	0.8	0.9
History of CHD or stroke	0.1	0.1	0.6	0.5	1.0
History of diabetes	0.2	0.2	0.3	0.3	0.4
25(OH)D status	4.7	4.7	4.9	4.9	4.7
Total cholesterol status	0.2	0.2	0.3	0.2	0.2
Crea-eGFR status	1.8	1.8	2.0	2.0	1.8
Multivariable model two[c, d]	11.1	11.2	12.5	12.2	12.1
Educational level	4.2	4.2	5.2	4.9	4.6

25(OH)D, 25-hydroxyvitamin D; AF, atrial fibrillation; CHD, coronary heart disease; Crea-eGFR, creatinine-estimated glomerular filtration rate; CVD, cardiovascular disease; HF, heart failure

[a] Restricted to Monitoring of Trends and Determinants in Cardiovascular disease (MONICA) Northern Sweden, FINRISK 1997, Scottish Heart Health Extended Cohort (SHHEC), and Moli-sani

[b] Restricted to MONICA Northern Sweden, FINRISK 1997, SHHEC, Moli-sani, and MONICA-Catalonia (cohort 1)

[c] The cohort-specific range of missing data was as follows for the largest population (*n* = 79,570):
Body mass index—0.0 (FINRISK 1997) to 1.1% (MONICA Brianza)
Smoking—0.0 (FINRISK 1997, SHHEC, and MONICA-Catalonia) to 1.0% (Moli-sani)
Systolic blood pressure—0.0 (FINRISK 1997, SHHEC, Moli-sani, and MONICA-Catalonia) to 0.9% (MONICA Brianza)
Antihypertensive medication—0.1 (SHHEC, MONICA/KORA, and Malattie Aterosclerotiche Istituto Superiore di Sanità [MATISS]) to 1.9% (FINRISK 1997)
CHD or stroke—0.0 (MONICA Northern Sweden and FINRISK 1997) to 3.7% (MONICA Brianza)
Diabetes—0.0 (MONICA Northern Sweden, FINRISK 1997, SHHEC, MONICA/ Cooperative Health Research in the Region of Augsburg [KORA], and MONICA-Catalonia) to 3.7% (MONICA Brianza)
25(OH)D—0.8 (MONICA Northern Sweden) to 11.3% (SHHEC)
Total cholesterol—0.0 (FINRISK 1997, SHHEC, and MONICA/KORA) to 0.6% (Moli-sani)
Crea-eGFR—0.0 (MONICA/KORA) to 3.8% (FINRISK 1997)
Education—0.1 (MONICA Brianza) to 100% (MATISS)

[d] The same variables as in model one with the addition of educational level. MATISS and subcohort 4 of MONICA/KORA were excluded, since data on educational level were not available in these cohorts
