# Supplementary material for: Cardiovascular disease outcomes in relation to 25-hydroxyvitamin D and its seasonal variation: Results from the BiomarCaRE consortium
Source: PLoS One. 2025 Apr 24;20(4):e0319607. doi: 10.1371/journal.pone.0319607 (PMC12021148; doi:10.1371/journal.pone.0319607)
Supplement: S4 Table — (PDF) [file pone.0319607.s007.pdf]

Characteristics[a]	Cohort							
	MONICA Northern Sweden	FINRISK 1997	SHHEC	MONICA/KORA	MONICA Brianza	Moli-sani	MATISS	MONICA-Catalonia
No. of participants	10,488	8002	14,902	8393	4781	24,243	3519	5242
Age (median, y)	48.8	48.4	49.8	50.2	46.6	54.6	50.5	45.7
Male sex (%)	49.4	50.0	50.2	49.7	49.3	48.1	34.3	54.3
Year of sampling (range)	1986–2009	1997	1984–1995	1994–2001	1986–1994	2005–2010	1993–1996	1986–1992
Latitude of habitat (range, °N)[b]	63.8–65.6	60.2–65.0	55.1–58.2	48.4	45.6	41.6	41.5	41.5
December-to-May sampling (%)	100	100	48.9	63.2	47.8	53.1	47.3	51.6
Highest third of education (%)[c]	35.4	38.4	28.6	36.7	36.9	42.7	—	36.1
BMI ≥ 30 kg/m^2 (%)	23.1	20.4	13.9	22.1	13.0	30.1	29.9	17.1
Daily smoker (%)[d]	24.6	26.6	44.8	27.0	32.3	22.7	21.7	39.7
Comorbidities (%)								
Cardiovascular disease[e]	5.2	5.8	4.1	3.4	1.6	2.8	3.0	2.3
Hypertension[f]	35.4	45.8	37.1	39.5	34.9	56.2	51.7	17.1
Diabetes	3.9	5.8	1.7	5.0	2.7	6.5	4.5	4.0
Biomarkers (median)								
25(OH)D (nmol/L)	45.2	32.4	36.4	43.4	40.9	43.7	42.9	33.4
Total cholesterol (mmol/L)	5.8	5.4	6.2	5.8	5.5	5.5	5.6	5.4
Crea-eGFR (ml/min/1.73 m^2)	103.3	89.4	95.9	98.0	92.1	94.3	98.9	109.5

25(OH)D, 25-hydroxyvitamin D; BMI, body mass index; Crea-eGFR, creatinine-estimated glomerular filtration rate; KORA, Cooperative Health Research in the Region of Augsburg; MATISS, Malattie Aterosclerotiche Istituto Superiore di Sanità; MONICA, Monitoring of Trends and Determinants in Cardiovascular disease; SHHEC, Scottish Heart Health Extended Cohort

[a] Based on complete data (see S3 Table for cohort-specific percentages of missing data)

[b] Based on the location of the largest city in each cohort or subcohort and retrieved from Google Maps (www.google.com/maps), except for in the SHHEC where district-level data on latitude were available

[c] Categories of education were derived from population-, sex-, and birth cohort-specific thirds of the distribution of years of education. No data on education were available in MATISS and in subcohort 4 of MONICA/KORA

[d] Cigarettes, cigars, cigarillos, or pipe

[e] Coronary heart disease or stroke

[f] Systolic blood pressure > 140 mmHg, diastolic blood pressure > 90 mmHg, or use of antihypertensive medication
