# Supplementary material for: Cardiovascular disease outcomes in relation to 25-hydroxyvitamin D and its seasonal variation: Results from the BiomarCaRE consortium
Source: PLoS One. 2025 Apr 24;20(4):e0319607. doi: 10.1371/journal.pone.0319607 (PMC12021148; doi:10.1371/journal.pone.0319607)
Supplement: S5 Table — The numbers within parenthesis (in smaller font) refer to the total number of cases in each strata. (PDF) [file pone.0319607.s008.pdf]

	Cohort-specific rates[a]															
	MONICA Northern Sweden		FINRISK 1997		SHHEC		MONICA/ KORA		MONICA Brianza		Moli-sani		MATISS		MONICA-Catalonia	
CVD endpoint	♂	♀	♂	♀	♂	♀	♂	♀	♂	♀	♂	♀	♂	♀	♂	♀
CHD[b]																
1990s	164 (67)	67 (31)	119 (26)	55 (10)	141 (274)	65 (135)	85 (17)	26 (5)	108 (44)	32 (14)	—	—	91 (11)	8 (2)	63 (30)	33 (12)
2000s	143 (109)	39 (34)	136 (100)	42 (36)	133 (263)	59 (137)	76 (54)	17 (13)	64 (29)	19 (10)	96 (54)	22 (13)	77 (9)	20 (5)	—	—
Stroke[b]																
1990s	77 (34)	51 (24)	72 (17)	22 (4)	50 (105)	24 (52)	43 (8)	30 (5)	35 (15)	11 (5)	—	—	40 (5)	24 (6)	4 (2)	5 (2)
2000s	65 (54)	42 (37)	60 (48)	22 (19)	40 (87)	23 (56)	45 (29)	25 (17)	27 (13)	13 (7)	8 (5)	3 (2)	16 (2)	8 (2)	—	—
Heart failure[b]																
1990s	52 (24)	33 (16)	44 (10)	28 (5)	57 (123)	41 (90)	—	—	—	—	—	—	—	—	—	—
2000s	34 (29)	22 (20)	82 (63)	60 (51)	52 (115)	27 (67)	—	—	—	—	73 (45)	65 (40)	—	—	—	—
Atrial fibrillation[b]																
1990s	57 (26)	33 (16)	50 (12)	26 (5)	41 (90)	21 (45)	—	—	—	—	—	—	—	—	5 (1)	9 (2)
2000s	55 (46)	35 (31)	68 (55)	42 (37)	64 (140)	27 (65)	—	—	—	—	58 (35)	23 (14)	—	—	—	—
CVD mortality																
1990s	62 (29)	29 (14)	52 (13)	21 (4)	98 (217)	38 (84)	56 (12)	10 (2)	54 (25)	9 (4)	—	—	47 (6)	8 (2)	38 (20)	18 (7)
2000s	43 (38)	20 (18)	57 (48)	16 (14)	72 (164)	37 (92)	43 (33)	9 (7)	12 (6)	7 (4)	3 (2)	3 (2)	24 (3)	12 (3)	—	—

CVD, cardiovascular disease; CHD, coronary heart disease; KORA, Cooperative Health Research in the Region of Augsburg; MATISS, Malattie Aterosclerotiche Istituto Superiore di Sanità*;* MONICA, Monitoring of Trends and Determinants in Cardiovascular disease; SHHEC, Scottish Heart Health Extended Cohort

[a] Estimated from a Poisson regression model, in which each observation was split by decade (1980s, 1990s, 2000s, 2010s) and attained age (ten-year age groups). The output is restricted to two decades and one age group

[b] Incidence rate
