# Supplementary material for: Cardiovascular disease outcomes in relation to 25-hydroxyvitamin D and its seasonal variation: Results from the BiomarCaRE consortium
Source: PLoS One. 2025 Apr 24;20(4):e0319607. doi: 10.1371/journal.pone.0319607 (PMC12021148; doi:10.1371/journal.pone.0319607)
Supplement: S6 Table — (PDF) [file pone.0319607.s009.pdf]

	Percentage with co-occurrence of CVD endpoints (*n*)[a]			
CVD endpoint (*n*)	Coronary heart disease	Stroke	Heart failure	Atrial fibrillation
Coronary heart disease (*n* = 3641)	—	10.2 (*n* = 371)	24.0 (*n* = 874)	16.9 (*n* = 616)
Stroke (*n* = 1603)	23.1 (*n* = 371)	—	15.1 (*n* = 242)	19.7 (*n* = 315)
Heart failure (*n* = 2102)	41.6 (*n* = 874)	11.5 (*n* = 242)	—	30.3 (*n* = 636)
Atrial fibrillation (*n* = 2051)	30.0 (*n* = 616)	15.4 (*n* = 315)	31.0 (*n* = 636)	—

CVD, cardiovascular disease

[a] Restricted to (i) Monitoring of Trends and Determinants in Cardiovascular disease (MONICA) Northern Sweden, FINRISK 1997, Scottish Heart Health Extended Cohort, and Moli-sani (since these cohort had complete baseline and follow-up data on each CVD endpoint) and (ii) participants who had no history of any CVD endpoint at the start of follow-up (*n* = 53,357). For this analysis, the follow-up period in Moli-sani ended on December 31, 2011 (since coronary heart disease was only followed up until that date, while stroke, heart failure, and atrial fibrillation had been followed up until December 31, 2015)
