# Supplementary material for: Cardiovascular disease outcomes in relation to 25-hydroxyvitamin D and its seasonal variation: Results from the BiomarCaRE consortium
Source: PLoS One. 2025 Apr 24;20(4):e0319607. doi: 10.1371/journal.pone.0319607 (PMC12021148; doi:10.1371/journal.pone.0319607)
Supplement: S7 Table — (PDF) [file pone.0319607.s010.pdf]

Calendar period	Rate ratio (95% CI)[a] CVD incidence	CVD mortality
Three-month[b, c]		
July, Aug, Sep	1.00 (reference)	1.00 (reference)
June, Oct, Nov	1.12 (1.04 to 1.20)	1.12 (1.01 to 1.24)
May, Dec, Jan	1.12 (1.04 to 1.20)	1.19 (1.08 to 1.32)
Feb, Mar, April	1.11 (1.03 to 1.19)	1.22 (1.10 to 1.36)
Three-month[d]		
June, July, Aug	1.00 (reference)	1.00 (reference)
Sep, Oct, Nov	1.08 (1.01 to 1.16)	1.03 (0.93 to 1.14)
Dec, Jan, Feb	1.10 (1.02 to 1.18)	1.20 (1.08 to 1.33)
Mar, April, May	1.09 (1.01 to 1.17)	1.12 (1.01 to 1.24)
Four-month[b, e]		
July, Aug, Sep, Oct	1.00 (reference)	1.00 (reference)
June, Nov, Dec, Jan	1.11 (1.04 to 1.18)	1.14 (1.05 to 1.25)
Feb, Mar, April, May	1.09 (1.03 to 1.16)	1.13 (1.04 to 1.24)
Six-month[b, f]		
June to Nov	1.00 (reference)	1.00 (reference)
Dec to May	1.05 (1.00 to 1.11)	1.14 (1.06 to 1.23)

CVD, cardiovascular disease

[a] Estimated from Poisson regression models and adjusted for sex, attained age, and cohort

[b] Ordered by the median regression-derived 25-hydroxyvitamin D (25[OH]D) concentration in the data (sex- and age-adjusted ranking, from highest to lowest: August, September, July, October, November, June, December, January, May, February, March, April)

[c] The ratio of the median 25(OH)D concentration was 1.00 (July, Aug, Sep; reference), 1.25 (June, Oct, Nov), 1.52 (May, Dec, Jan), and 1.71 (Feb, Mar, April)

[d] Ordered by traditional calendar seasons: summer (June, July, Aug), autumn (Sep, Oct, Nov), winter (Dec, Jan, Feb), and spring (Mar, April, May)

[e] The ratio of the median 25(OH)D concentration was 1.00 (July, Aug, Sep, Oct; reference), 1.37 (June, Nov, Dec, Jan), and 1.63 (Feb, Mar, April, May)

[f] The ratio of the median 25(OH)D concentration was 1.00 (June to Nov; reference) and 1.44 (Dec to May)
