# Supplementary material for: Cardiovascular disease outcomes in relation to 25-hydroxyvitamin D and its seasonal variation: Results from the BiomarCaRE consortium
Source: PLoS One. 2025 Apr 24;20(4):e0319607. doi: 10.1371/journal.pone.0319607 (PMC12021148; doi:10.1371/journal.pone.0319607)
Supplement: S8 Table — (PDF) [file pone.0319607.s011.pdf]

	Two-month calendar period (ordered by increasing differences in 25[OH]D concentration)[a]					
CVD endpoint	August, September	July, October	June, November	January, December	February, May	March, April
Coronary heart disease						
No. of cases/person-years	736/142,406	751/144,927	813/140,373	797/142,567	778/135,255	744/139,839
Rate ratio (95% CI)[b]	1.00 (reference)	1.00 (0.91 to 1.11)	1.12 (1.02 to 1.24)	1.08 (0.98 to 1.20)	1.12 (1.02 to 1.24)	1.04 (0.94 to 1.15)
Stroke						
No. of cases/person-years	372/160,407	363/163,229	411/158,085	398/160,798	367/152,617	434/157,703
Rate ratio (95% CI)[b]	1.00 (reference)	0.96 (0.83 to 1.11)	1.12 (0.98 to 1.29)	1.07 (0.93 to 1.24)	1.05 (0.91 to 1.21)	1.20 (1.05 to 1.38)

25(OH)D, 25-hydroxyvitamin D; CVD, cardiovascular disease

[a] The ratio of the median 25(OH)D concentration was 1.00 (August, September; reference), 1.10 (July, October), 1.37 (June, November), 1.54 (January, December), 1.66 (February, May), and 1.79 (March, April)

[b] Estimated from Poisson regression models and adjusted for sex, attained age, and cohort
