# Supplementary material for: Cardiovascular disease outcomes in relation to 25-hydroxyvitamin D and its seasonal variation: Results from the BiomarCaRE consortium
Source: PLoS One. 2025 Apr 24;20(4):e0319607. doi: 10.1371/journal.pone.0319607 (PMC12021148; doi:10.1371/journal.pone.0319607)
Supplement: S9 Table — (PDF) [file pone.0319607.s012.pdf]

CVD endpoint	Quarters of 25(OH)D concentration (nmol/L)[a] One (lowest)	Two	Three	Four (highest)
Coronary heart disease[b]				
No. of cases/person-years	1178/191,384	1122/195,309	978/199,215	897/196,075
HR (95% CI)	1.00 (reference)	0.97 (0.89 to 1.05)	0.86 (0.79 to 0.94)	0.82 (0.75 to 0.90)
Stroke[b]				
No. of cases/person-years	605/216,802	550/220,068	513/224,829	470/220,300
HR (95% CI)[c]	1.00 (reference)	0.92 (0.82 to 1.03)	0.85 (0.75 to 0.96)	0.80 (0.71 to 0.91)
Heart failure[b]				
No. of cases/person-years	926/161,470	737/164,262	649/168,156	592/165,409
HR (95% CI)[c]	1.00 (reference)	0.86 (0.78 to 0.95)	0.79 (0.71 to 0.88)	0.78 (0.70 to 0.87)
Atrial fibrillation[b]				
No. of cases/person-years	653/166,888	622/169,523	653/172,437	628/169,302
HR (95% CI)[c]	1.00 (reference)	0.98 (0.88 to 1.10)	1.01 (0.90 to 1.13)	1.02 (0.91 to 1.15)

25(OH)D, 25-hydroxyvitamin D; CVD, cardiovascular disease; HR, hazard ratio

[a] Sex-, cohort- and calendar month-specific quarters

[b] The number of participants included in each analysis: 71,210 for coronary heart disease, 71,913 for stroke, 52,485 for heart failure, and 54,418 for atrial fibrillation

[c] Estimated from Cox regression models and adjusted for the same variables as in Table 4
