# Supplementary material for: Cardiovascular disease outcomes in relation to 25-hydroxyvitamin D and its seasonal variation: Results from the BiomarCaRE consortium
Source: PLoS One. 2025 Apr 24;20(4):e0319607. doi: 10.1371/journal.pone.0319607 (PMC12021148; doi:10.1371/journal.pone.0319607)
Supplement: S10 Table — (PDF) [file pone.0319607.s013.pdf]

Sensitivity analysis of Cox regression models	No. of cases/ person-years	Quarters of 25(OH)D concentration (nmol/L) One (lowest)	Two	Three	Four (highest)
Complete case data[a]					
HR (95% CI) for CVD incidence	5449/761,053	1.00 (reference)	0.98 (0.91 to 1.05)	0.86 (0.79 to 0.92)	0.81 (0.75 to 0.88)
HR (95% CI) for CVD mortality	2619/912,717	1.00 (reference)	0.90 (0.82 to 1.00)	0.74 (0.66 to 0.83)	0.63 (0.56 to 0.71)
Complete case data, adjusted for education[b]					
HR (95% CI) for CVD incidence	5018/683,200	1.00 (reference)	0.98 (0.91 to 1.06)	0.86 (0.80 to 0.93)	0.81 (0.75 to 0.88)
HR (95% CI) for CVD mortality	2397/818,799	1.00 (reference)	0.92 (0.83 to 1.02)	0.74 (0.66 to 0.83)	0.64 (0.56 to 0.72)
Complete case data, restricted follow-up time[c]					
HR (95% CI) for CVD incidence	1482/320,914	1.00 (reference)	0.93 (0.82 to 1.07)	0.84 (0.73 to 0.97)	0.78 (0.67 to 0.91)
HR (95% CI) for CVD mortality	531/359,699	1.00 (reference)	0.76 (0.60 to 0.95)	0.61 (0.48 to 0.79)	0.62 (0.48 to 0.81)
Complete case data, re-categorized exposure[d]					
HR (95% CI) for CVD incidence	5449/761,053	1.00 (reference)	0.95 (0.88 to 1.02)	0.85 (0.79 to 0.92)	0.81 (0.75 to 0.88)
HR (95% CI) for CVD mortality	2619/912,717	1.00 (reference)	0.85 (0.77 to 0.94)	0.73 (0.65 to 0.81)	0.63 (0.56 to 0.70)

25(OH)D, 25-hydroxyvitamin D; CVD, cardiovascular disease; HR, hazard ratio

[a] Sex-, cohort- and calendar month-specific quarters. Based on complete data in all cohorts and adjusted for the same variables as in Table 4 (eligible for analysis: 69,829 participants for CVD incidence and 73,488 participants for CVD mortality)

[b] Sex-, cohort- and calendar month-specific quarters. Based on complete data in all cohorts (excluding Malattie Aterosclerotiche Istituto Superiore di Sanità [MATISS] and subcohort 4 of Monitoring of Trends and Determinants in Cardiovascular Disease [MONICA]/Cooperative Health Research in the Region of Augsburg [KORA]) and adjusted for the same variables as in Table 4 as well as for educational level (eligible for analysis: 60,455 participants for CVD incidence and 63,326 participants for CVD mortality)

[c] Sex-, cohort- and calendar month-specific quarters. Based on complete data in all cohorts and adjusted for the same variables as in Table 4 (eligible for analysis: 69,829 participants for CVD incidence and 73,488 participants for CVD mortality). Compared to the main model, the time-at-risk was set to a maximum of 5 years

[d] Sex-, subcohort- and calendar month-specific quarters. Based on complete data in all cohorts and adjusted for the same variables as in Table 4, with the exception that the variable cohort (categorical, eight levels) was replaced with the variable subcohort (categorical, 14 levels) (eligible for analysis: 69,829 participants for CVD incidence and 73,488 participants for CVD mortality). See S1 Text for details on how the exposure variable was re-categorized
