# Supplementary material for: Cardiovascular disease outcomes in relation to 25-hydroxyvitamin D and its seasonal variation: Results from the BiomarCaRE consortium
Source: PLoS One. 2025 Apr 24;20(4):e0319607. doi: 10.1371/journal.pone.0319607 (PMC12021148; doi:10.1371/journal.pone.0319607)
Supplement: S11 Table — (PDF) [file pone.0319607.s014.pdf]

	HR for the highest vs the lowest quarter of 25(OH)D concentration[a]									
	Main model	Model one[b]			Model two[c]			Model three[d]		
Endpoint		1 year	2 year	3 year	1 year	2 year	3 year	1 year	2 year	3 year
CVD incidence	0.82	0.81	0.81	0.81	—	—	—	0.82	0.81	0.81
CVD mortality	0.64	0.64	0.66	0.66	0.67	0.68	0.68	0.65	0.66	0.67

25(OH)D, 25-hydroxyvitamin D; CVD, cardiovascular disease; HR, hazard ratio

[a] Based on multiple imputed data and estimated from Cox regression models that were adjusted for the same variables as in Table 4

[b] Delaying the start of follow-up with one, two, and three years (eligible for analysis: 74,344, 73,420, and 68,179 participants, respectively, for CVD incidence and 79,281, 78,888, and 76,789 participants, respectively, for CVD mortality)

[c] As Model one and further excluding participants with prevalent CVD at baseline ($n = 0$ and 3629 participants, respectively)

[d] As Model two and further excluding participants with N-terminal pro-B-type natriuretic peptide or high-sensitive troponin I concentrations above the 99th percentile of the disease-free population at baseline ($n = 1314$ and 1330 participants, respectively)
