# Supplementary material for: Cardiovascular disease outcomes in relation to 25-hydroxyvitamin D and its seasonal variation: Results from the BiomarCaRE consortium
Source: PLoS One. 2025 Apr 24;20(4):e0319607. doi: 10.1371/journal.pone.0319607 (PMC12021148; doi:10.1371/journal.pone.0319607)
Supplement: S1 Fig — Data from Sweden and Finland are not included in the country-specific analysis due to a non-full year examination period (winter-to-spring); however, full season data from Sweden and Finland are available elsewhere (Klingberg et al, Endocrine 49, 800–808; Stridh et al, Sci Rep 11, 20989). (PDF) [file pone.0319607.s015.pdf]

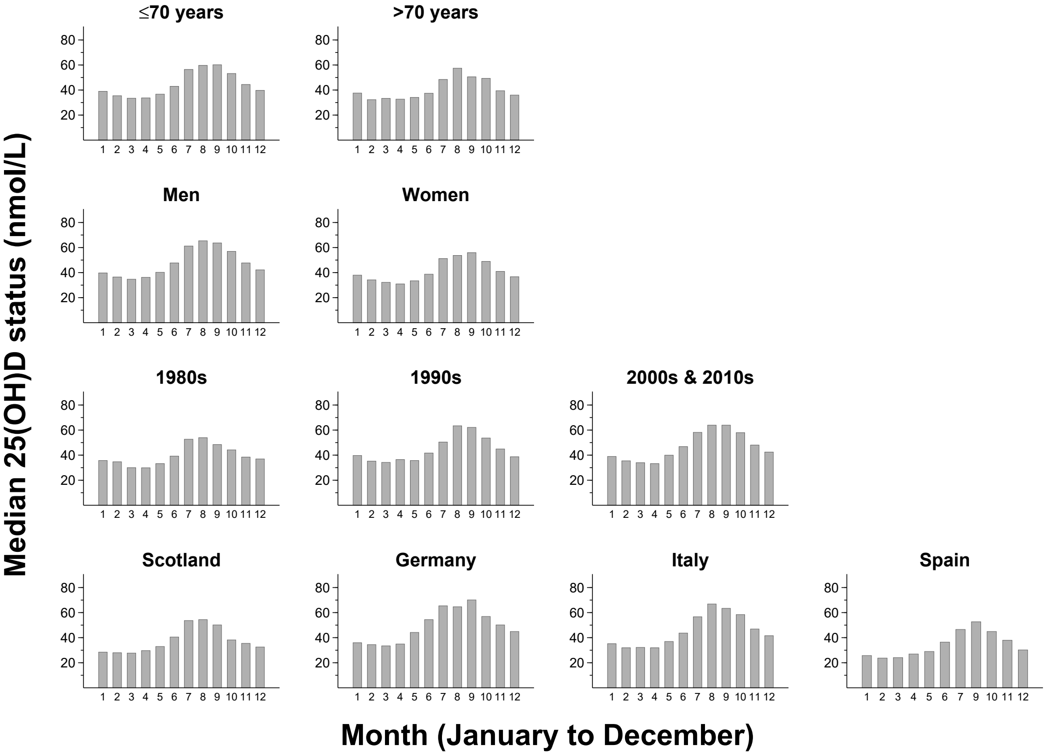
≤70 years
>70 years
Men
Women
1980s
1990s
2000s & 2010s
Scotland
Germany
Italy
Spain
80
60
40
20
1 2 3 4 5 6 7 8 9 10 11 12
1 2 3 4 5 6 7 9 10 11 12
Median 25(OH)D status (nmol/L)
Month (January to December)
