# Supplementary material for: Cardiovascular disease outcomes in relation to 25-hydroxyvitamin D and its seasonal variation: Results from the BiomarCaRE consortium
Source: PLoS One. 2025 Apr 24;20(4):e0319607. doi: 10.1371/journal.pone.0319607 (PMC12021148; doi:10.1371/journal.pone.0319607)
Supplement: S2 Fig — The solid lines represent the differences in median 25(OH)D concentrations and the dashed lines represent the 95% CI. Estimates were adjusted for sex and season of sampling (winter, spring, summer, and fall). The reference value was set to the median value of age in each cohort. (PDF) [file pone.0319607.s016.pdf]

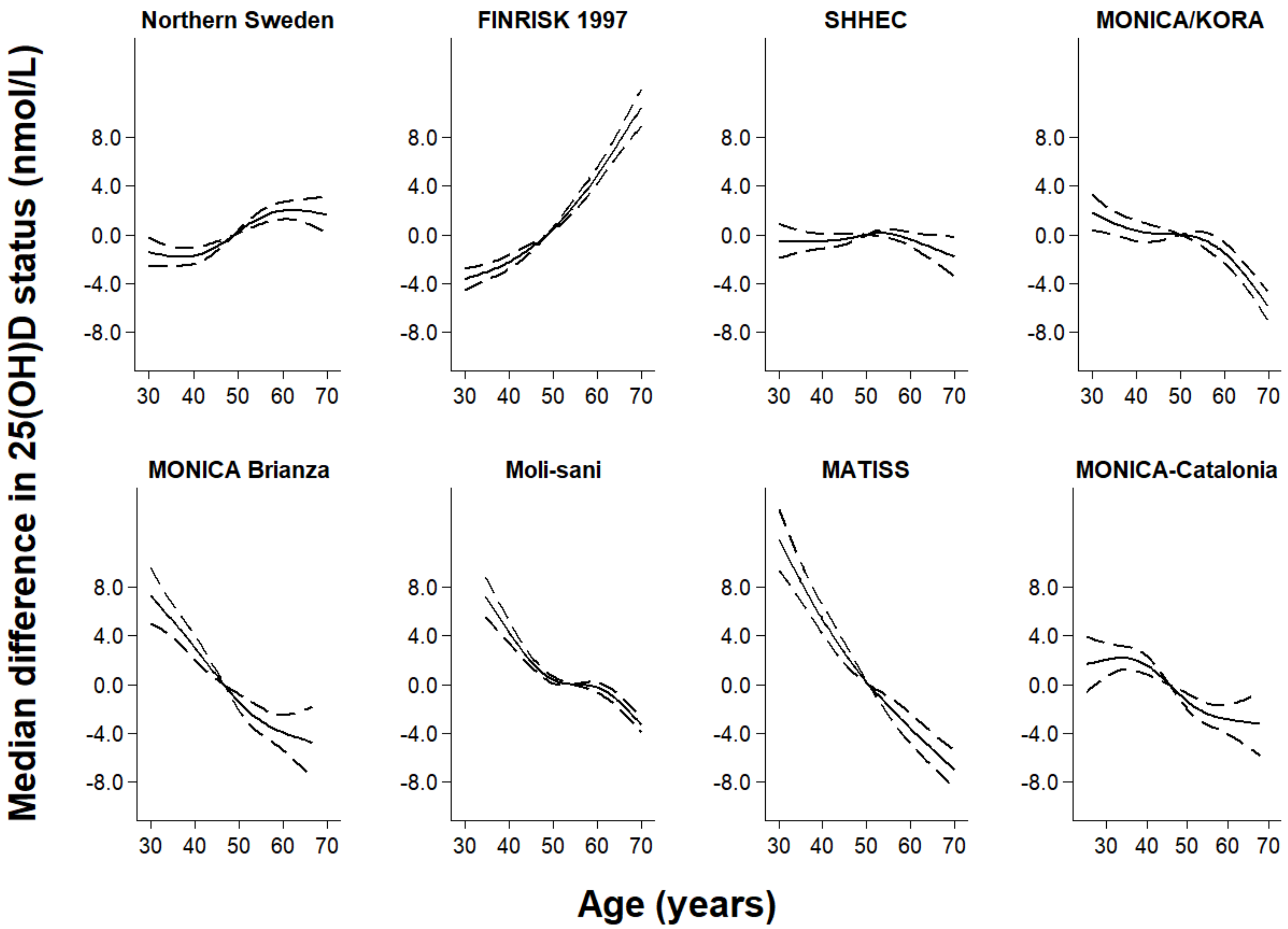

Abbreviations: KORA, Cooperative Health Research in the Region of Augsburg; MATISS, Malattie Aterosclerotiche Istituto Superiore di Sanità; MONICA, Monitoring of Trends and Determinants in Cardiovascular disease; SHHEC, Scottish Heart Health Extended Cohort
