# Supplementary figures and images for: Cardiovascular disease outcomes in relation to 25-hydroxyvitamin D and its seasonal variation: Results from the BiomarCaRE consortium
Source: PLoS One. 2025 Apr 24;20(4):e0319607. doi: 10.1371/journal.pone.0319607 (PMC12021148; doi:10.1371/journal.pone.0319607)

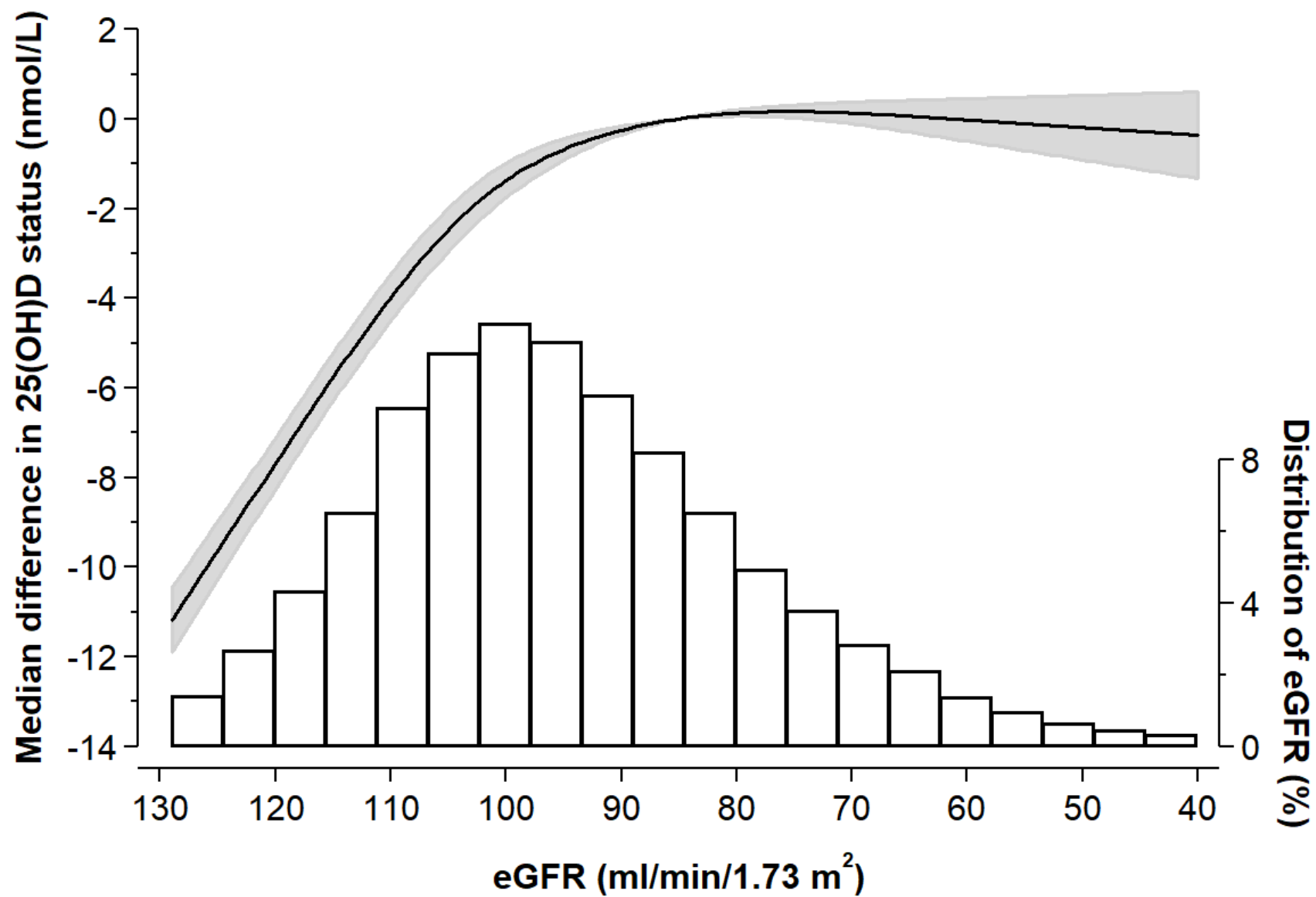

Supplement: S3 Fig — The solid line represents the difference in median 25(OH)D concentrations and the shaded area represents the 95% CI (on the left y-axis). The histogram represents the distribution of eGFR in the study population (on the right y-axis). Estimates were adjusted for sex, age (continuous using four-knot restricted cubic splines, years), and season of sampling (winter, spring, summer, and fall). The reference value was set to the median value of eGFR in the study population (85 ml/min/1.73 m2). (PDF) [file pone.0319607.s017.pdf]

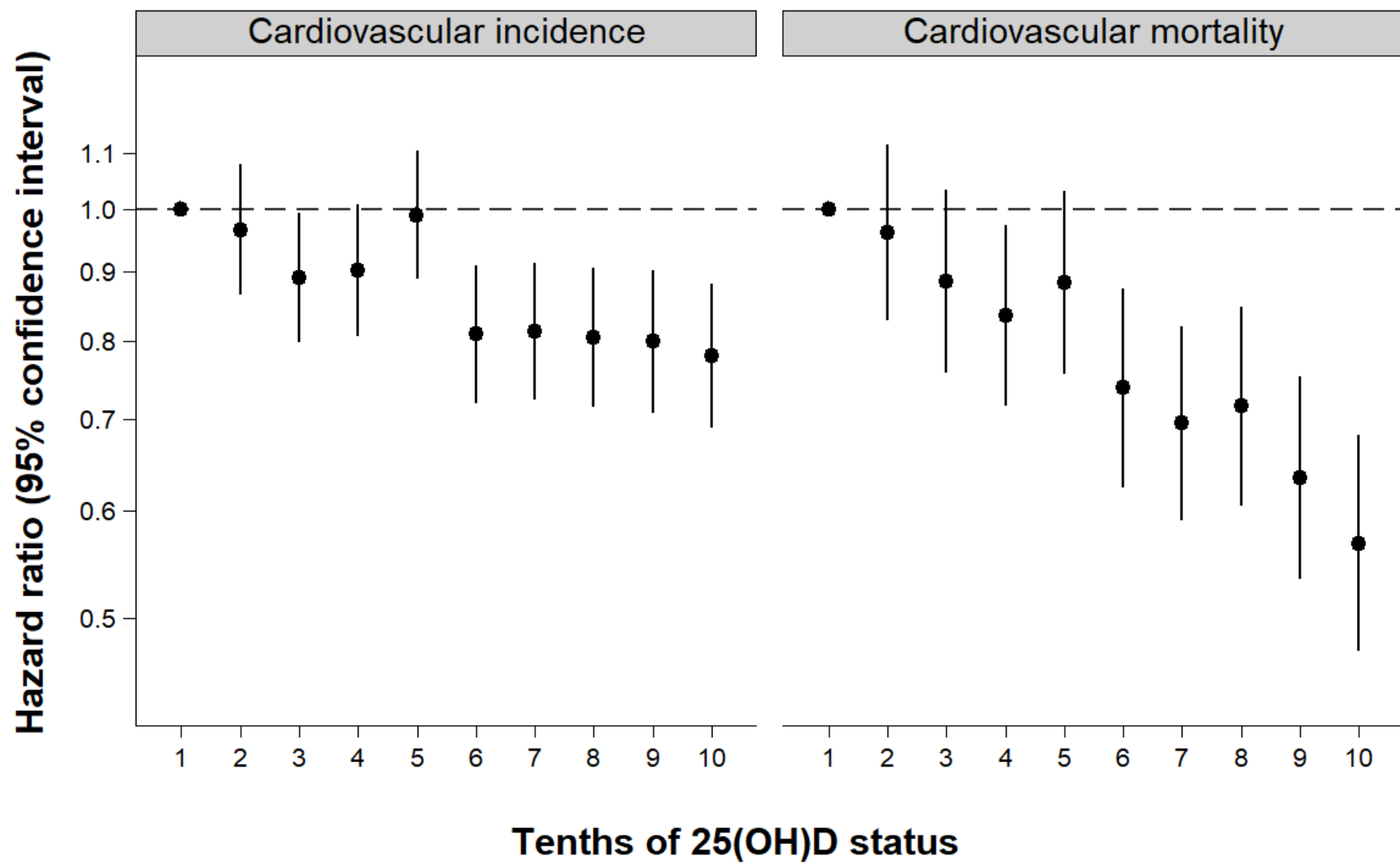

Supplement: S6 Fig — Circles and spikes represent point estimates and 95% CI, which were based on multiple imputed data, derived from Cox regression models, and adjusted for the same covariates as in Table 4. (PDF) [file pone.0319607.s020.pdf]

## Page A: Cardiovascular disease incidence

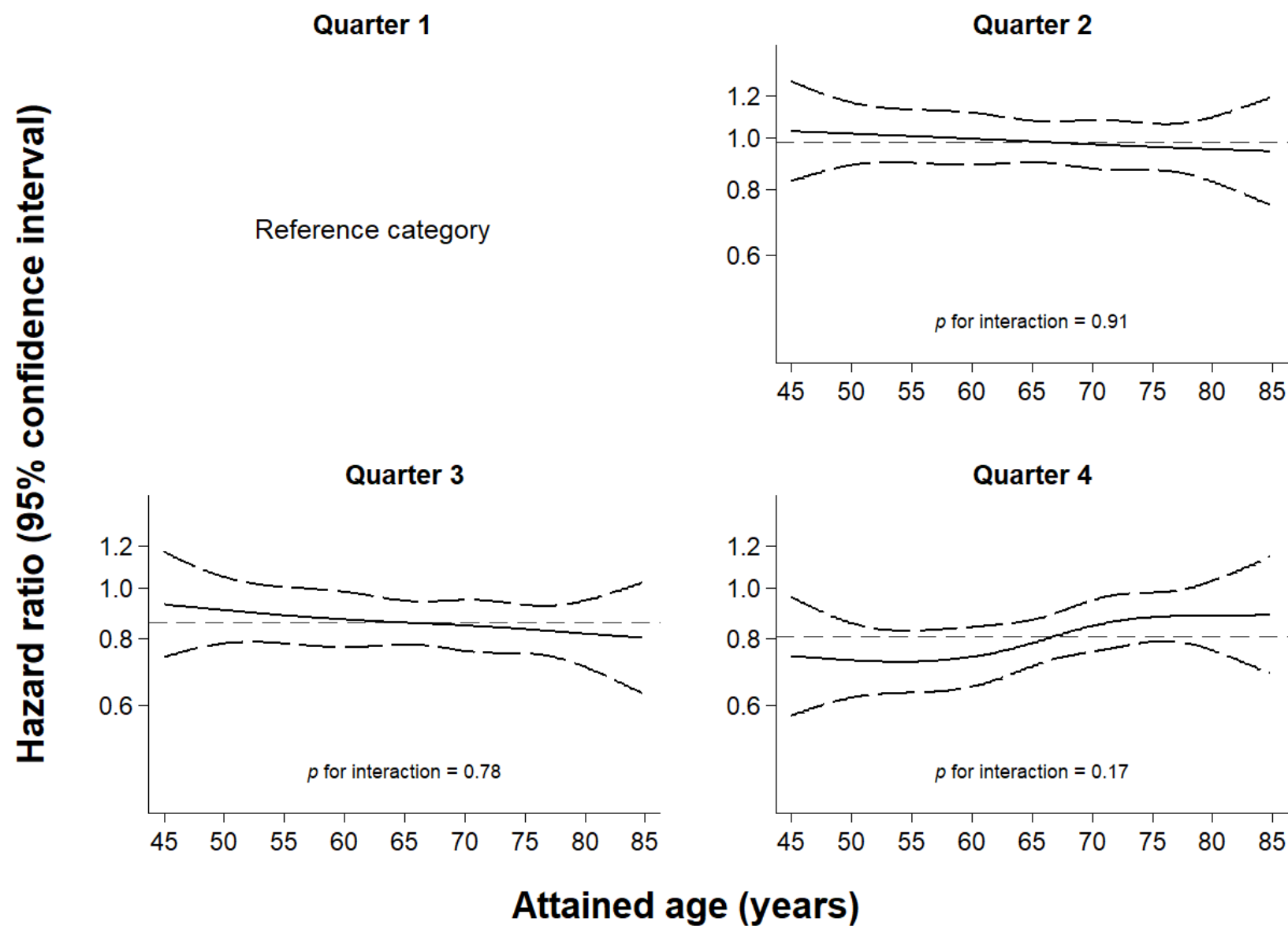

## Page B: Cardiovascular disease mortality

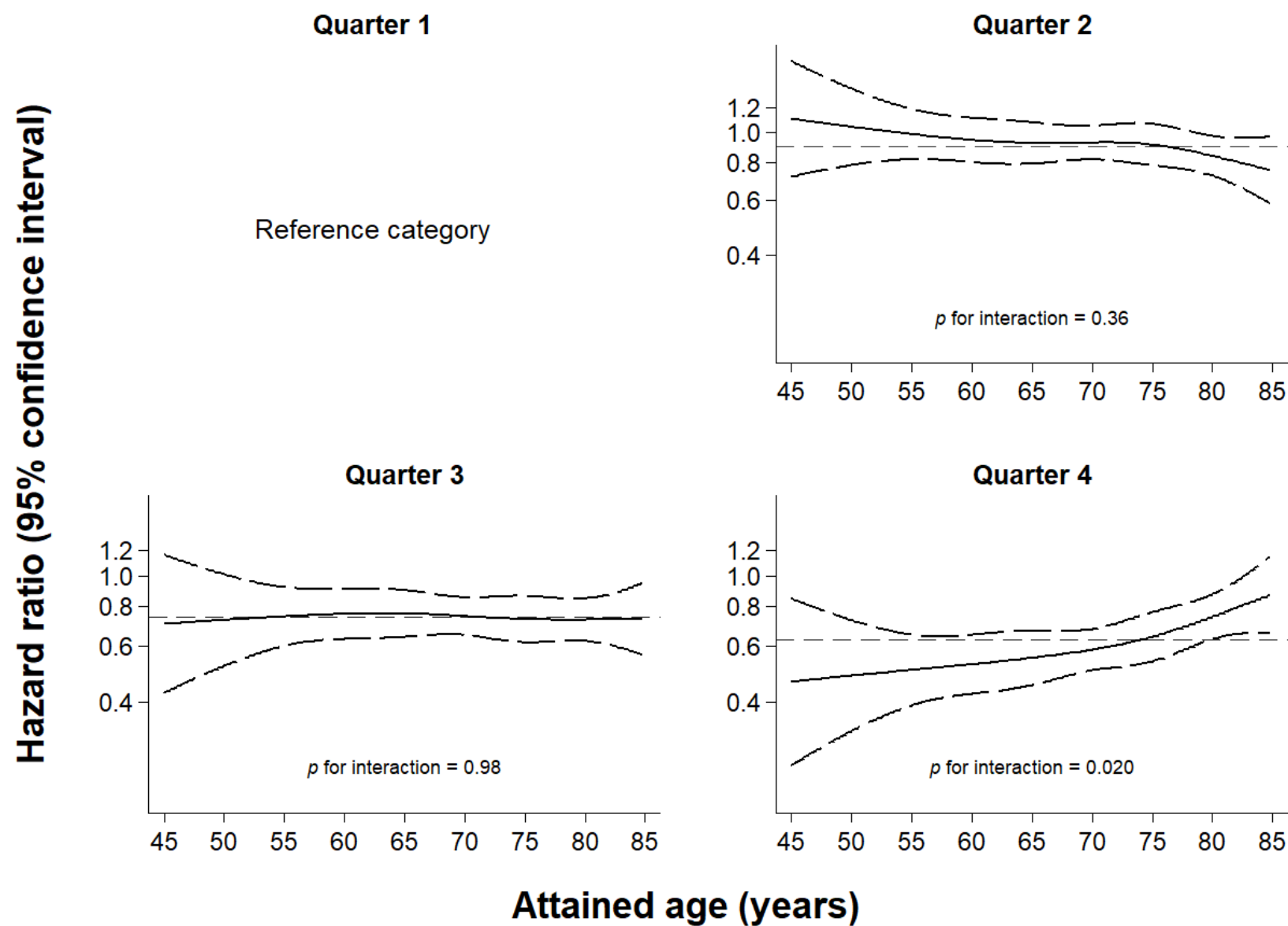

Supplement: S7 Fig — The solid and long dashed lines represent time-varying estimates and 95% CI, which were based on complete case data and derived from a Cox regression model that included an interaction term between 25(OH)D status (in quarters) and attained age (as a continuous variable, modeled using four-knots restricted cubic splines). The short dashed lines represent the time-fixed estimate based on complete case data. All estimates were adjusted for the same covariates as in Table 4. The reported p values for interaction were calculated by testing the second and third spline transformation jointly equal to zero. (PDF) [file pone.0319607.s021.pdf]

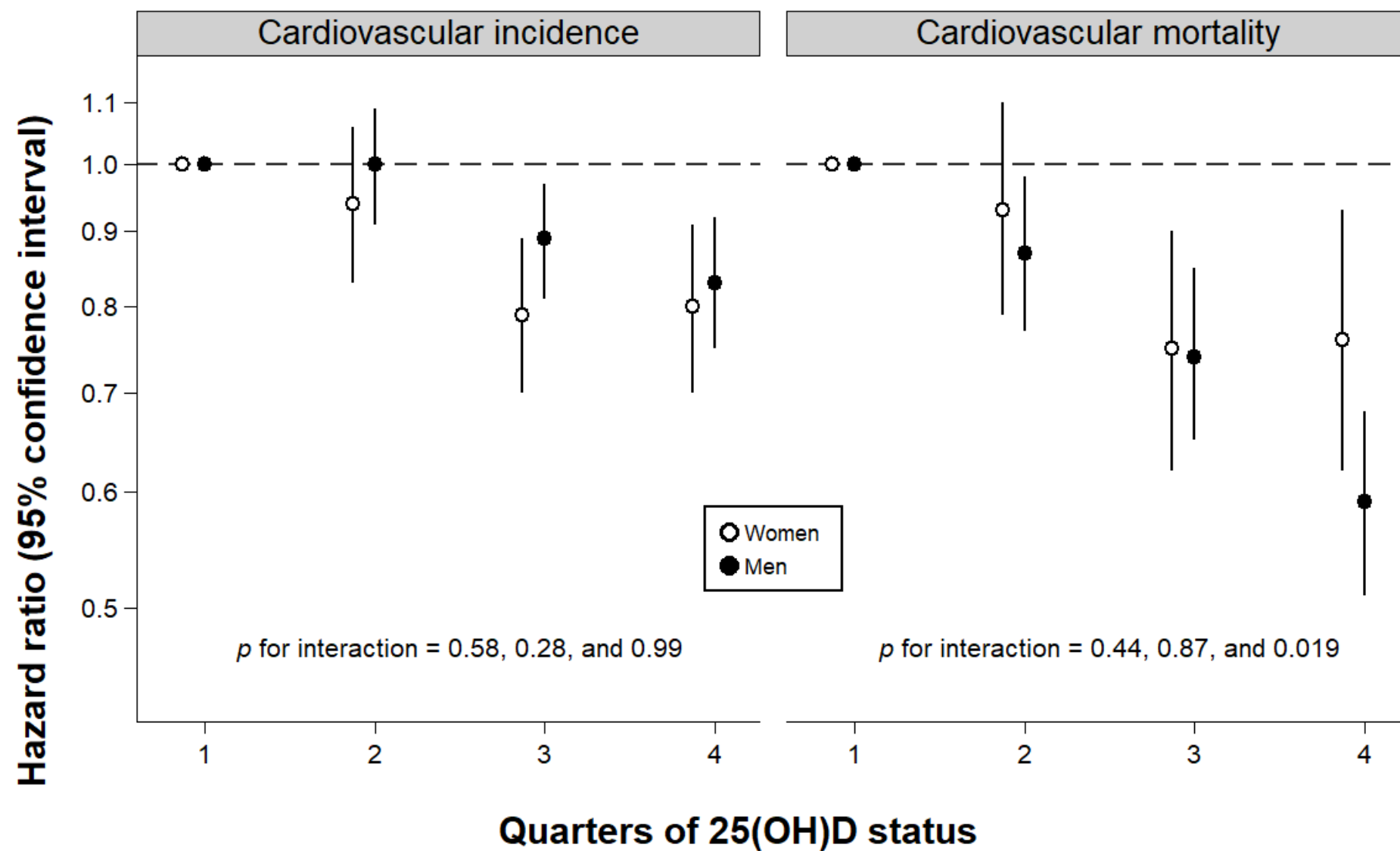

Supplement: S8 Fig — Circles and spikes represent point estimates and 95% CI, which were based on multiple imputed data, derived from Cox regression models, and adjusted for the same covariates as in Table 4. The reported p values for interaction were calculated by including an interaction tern between 25(OH)D status and sex in the Cox regression model and testing its coefficients equal to zero. The p value for an overall interaction (testing the coefficients jointly equal to zero) was 0.68 and 0.10, respectively, for cardiovascular disease incidence and mortality. (PDF) [file pone.0319607.s022.pdf]
