# Supplementary material for: Cardiovascular disease outcomes in relation to 25-hydroxyvitamin D and its seasonal variation: Results from the BiomarCaRE consortium
Source: PLoS One. 2025 Apr 24;20(4):e0319607. doi: 10.1371/journal.pone.0319607 (PMC12021148; doi:10.1371/journal.pone.0319607)
Supplement: S4 Fig — Circles and spikes represent point estimates and 95% CI, which were derived from Poisson regression models and adjusted for sex, attained age, and cohort. The reported p values for interaction were calculated by including an interaction term between the two-month calendar periods and 25(OH)D status in the Poisson regression model and testing its coefficients equal to zero. The p value for an overall interaction by testing the coefficients jointly equal to zero was 0.98 and 0.67, respectively, for cardiovascular disease incidence and mortality. (PDF) [file pone.0319607.s018.pdf]

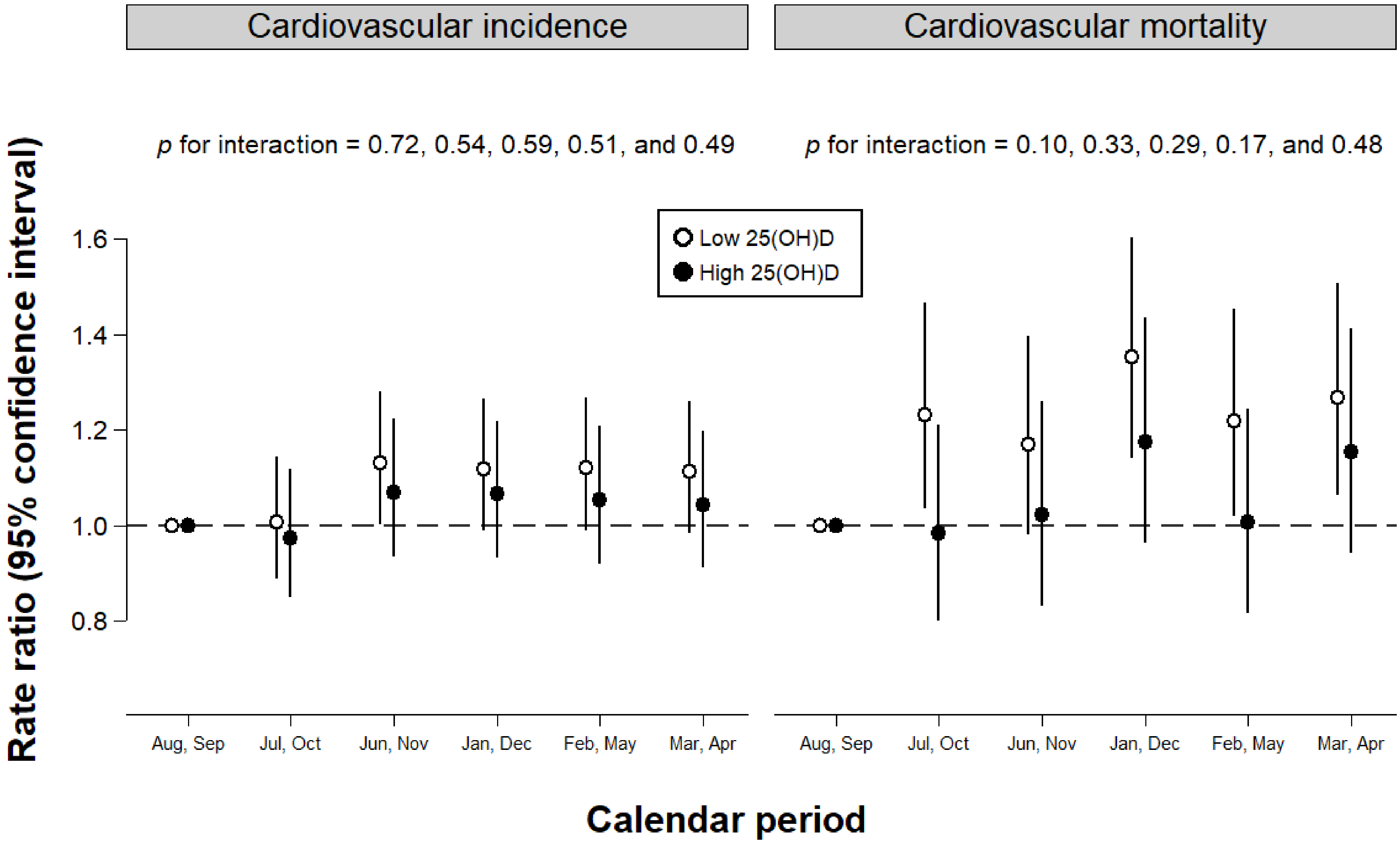
Cardiovascular incidence
Cardiovascular mortality
p for interaction = 0.72, 0.54, 0.59, 0.51, and 0.49
p for interaction = 0.10, 0.33, 0.29, 0.17, and 0.48
Low 25(OH)D
High 25(OH)D
Rate ratio (95% confidence interval)
1.6
1.4
1.2
1.0
0.8
Aug, Sep
Jul, Oct
Jun, Nov
Jan, Dec
Feb, May
Mar, Apr
Calendar period
