# Supplementary material for: Cardiovascular disease outcomes in relation to 25-hydroxyvitamin D and its seasonal variation: Results from the BiomarCaRE consortium
Source: PLoS One. 2025 Apr 24;20(4):e0319607. doi: 10.1371/journal.pone.0319607 (PMC12021148; doi:10.1371/journal.pone.0319607)
Supplement: S5 Fig — Circles and capped spikes represent point estimates and 95% CI, which were derived from Poisson regression models and adjusted for sex, attained age, and cohort (where appropriate). The p values refer to interaction effects (see S1 Text for details on how the tests for interaction were conducted). (PDF) [file pone.0319607.s019.pdf]

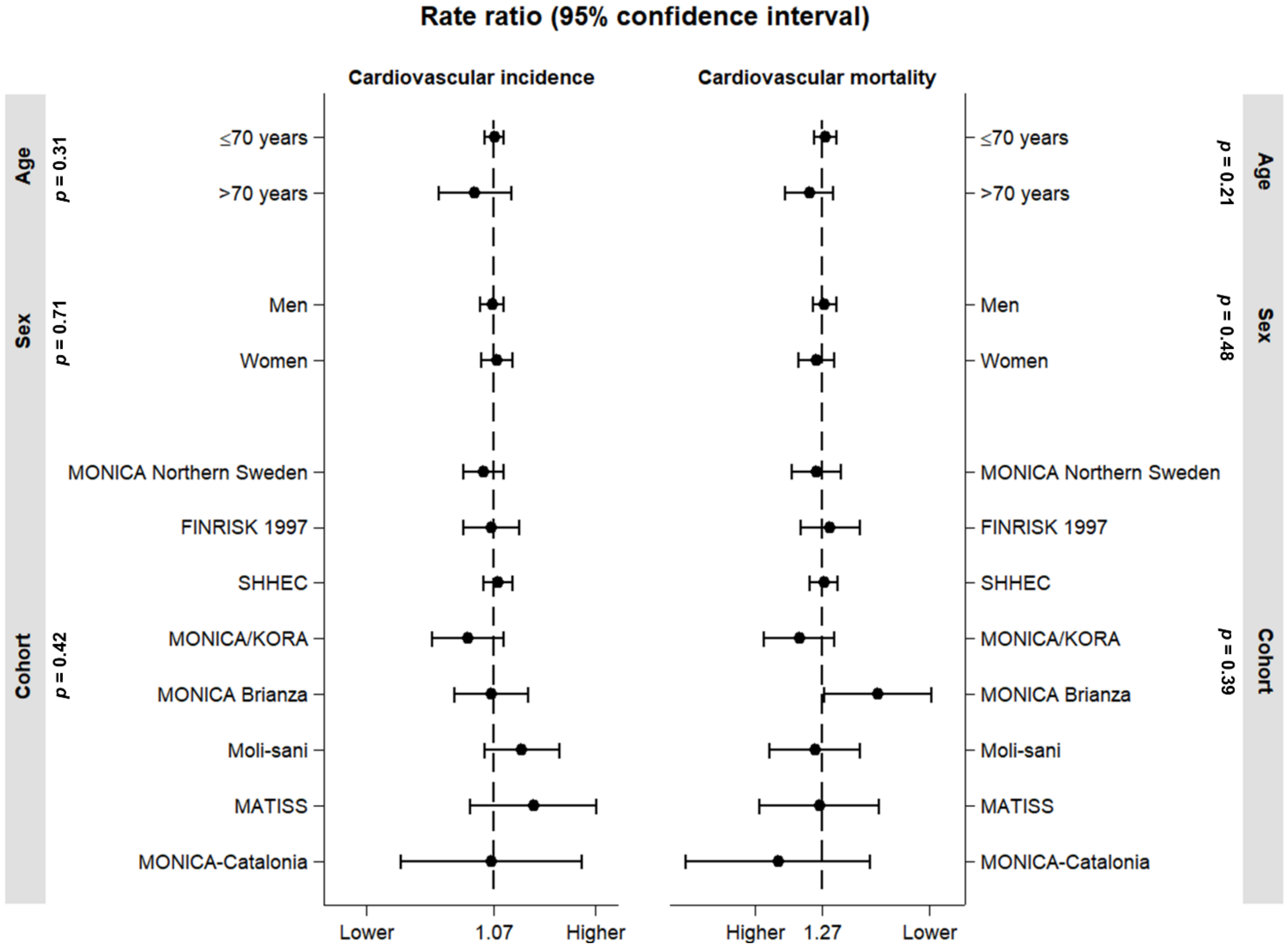

Abbreviations: KORA, Cooperative Health Research in the Region of Augsburg; MATISS, Malattie Aterosclerotiche Istituto Superiore di Sanità; MONICA, Monitoring of Trends and Determinants in Cardiovascular disease; SHHEC, Scottish Heart Health Extended Cohort
