# Supplementary material for: Cardiovascular disease outcomes in relation to 25-hydroxyvitamin D and its seasonal variation: Results from the BiomarCaRE consortium
Source: PLoS One. 2025 Apr 24;20(4):e0319607. doi: 10.1371/journal.pone.0319607 (PMC12021148; doi:10.1371/journal.pone.0319607)
Supplement: S9 Fig — HRs and 95% CI were based on multiple imputed data and derived from Cox regression models, which were adjusted for the same covariates as in Table 4. The dashed lines represent the point estimates in the pooled analysis. The reported p values for interaction were calculated using the Cochran Q test from a random-effects meta-analysis. (PDF) [file pone.0319607.s023.pdf]

Page A: Cardiovascular disease incidence

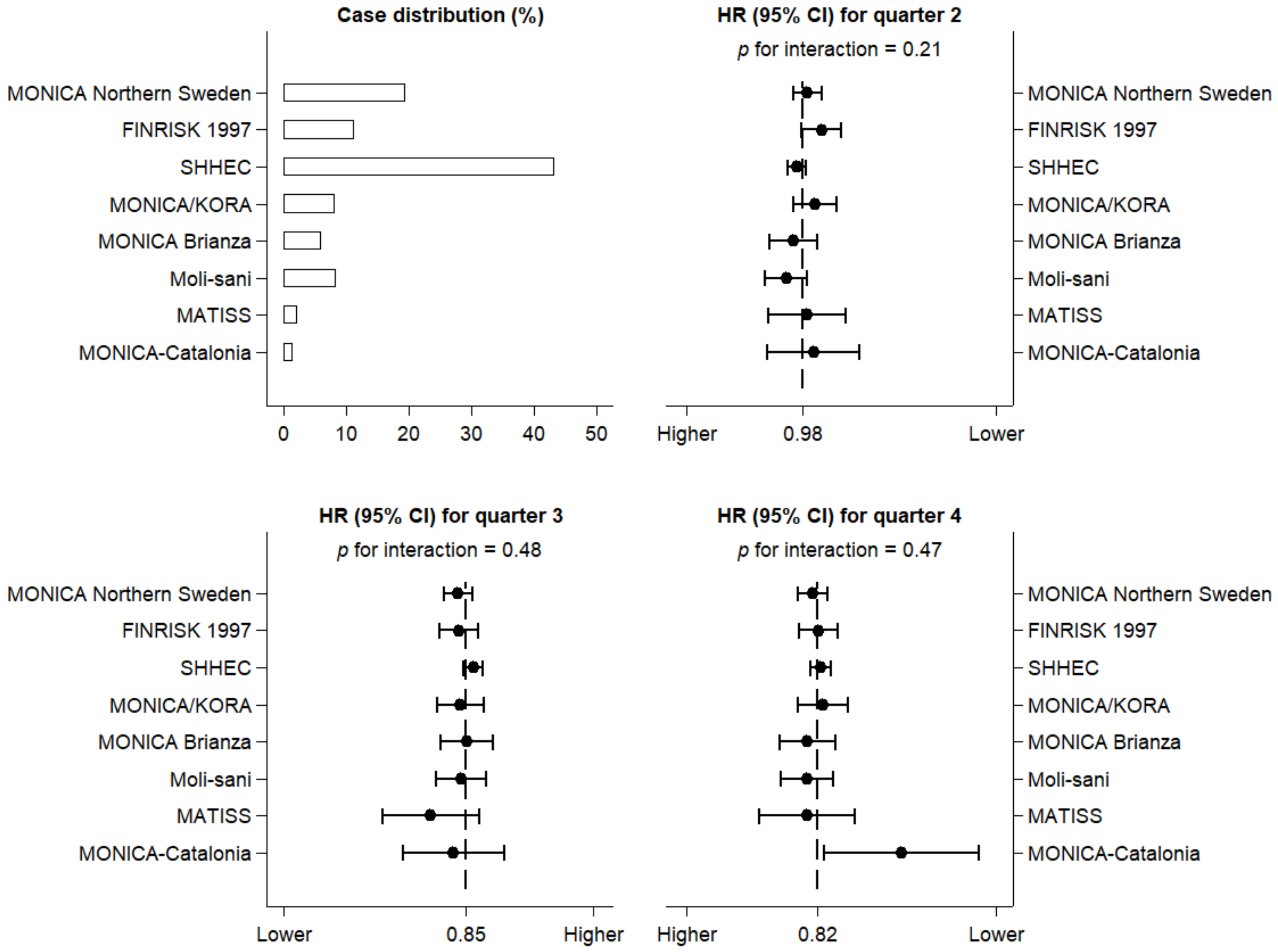

Abbreviations: KORA, Cooperative Health Research in the Region of Augsburg; MATISS, Malattie Aterosclerotiche Istituto Superiore di Sanità; MONICA, Monitoring of Trends and Determinants in Cardiovascular disease; SHHEC, Scottish Heart Health Extended Cohort

Page B: Cardiovascular disease mortality

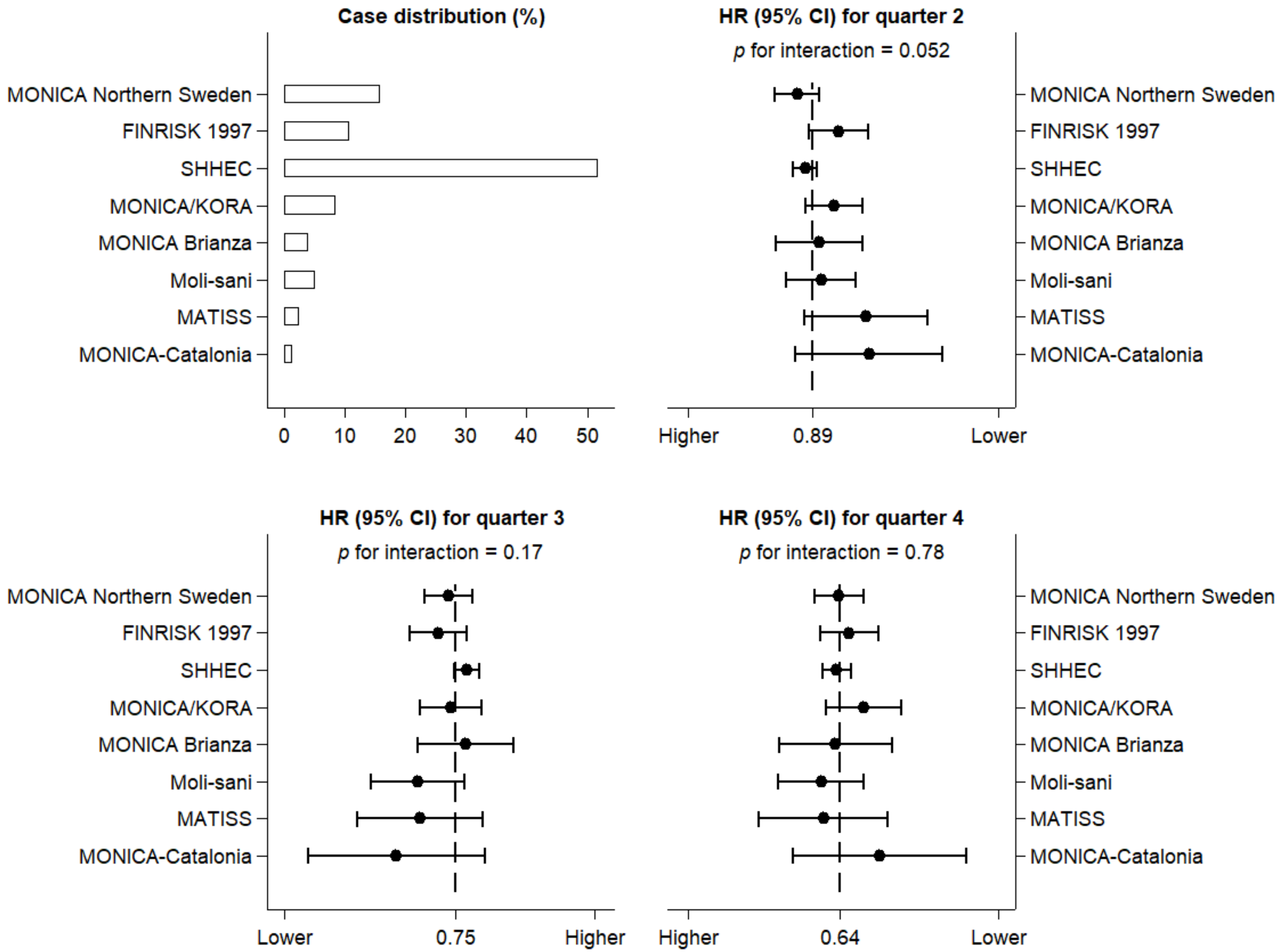

Abbreviations: KORA, Cooperative Health Research in the Region of Augsburg; MATISS, Malattie Aterosclerotiche Istituto Superiore di Sanità; MONICA, Monitoring of Trends and Determinants in Cardiovascular disease; SHHEC, Scottish Heart Health Extended Cohort
